# Supplementary material for: Environmental lead risk in the 21st century
Source: Commun Earth Environ. 2025 Sep 30;6(1):776. doi: 10.1038/s43247-025-02735-x (PMC12484081; doi:10.1038/s43247-025-02735-x)
Supplement: Supplementary file 2 — Supplementary Information for Environmental lead risk in the 21st century [file 43247_2025_2735_MOESM2_ESM.pdf]

Supplementary Information for

Environmental lead risk in the 21st century

Mengli Chen[1,*], Ludovica Gazze[2], Francis J. DiTraglia[3], Reshmi Das[4], Jerome Nriagu[5], Yigal Erel[6], Edward A. Boyle[7], Caroline M. Taylor[8], Dominik Weiss[9,*]

[1] Tropical Marine Science Institute, National University of Singapore, Singapore, Singapore

[2] Department of Economics, University of Warwick, Coventry, United Kingdom

[3] Department of Economics, University of Oxford, Oxford, United Kingdom

[4] School of Environmental Studies, Jadavpur University, Kolkata, India

[5] School of Public Health, University of Michigan, Ann Arbor, United States of America

[6] The Fredy and Nadine Herrmann Institute of Earth Sciences, Hebrew University of Jerusalem, Jerusalem, Israel

[7] Earth, Atmospheric and Planetary Science, Massachusetts Institute of Technology, Cambridge, Massachusetts, United States of America

[8] Centre for Academic Child Health, Bristol Medical School, University of Bristol, Bristol, United Kingdom

[9] Earth Science and Engineering, Imperial College London, London, United Kingdom

*Correspondence to d.weiss@imperial.ac.uk, mengli.chen@nus.edu.sg

Supplementary text 3

Text S1. History of leaded gasoline 3

Text S2. Lead in the atmospheric, marine, and terrestrial environment during the leaded gasoline period 5

Atmospheric environment 5

Marine environment 5

Terrestrial environment 6

Text S3. Methodologies in compiling and estimating the gasoline lead emission 7

Text S4. Methodologies in estimating lead exposure 10

Overview 10

Step 1: Approximation of the distribution of blood lead levels 10

Step 2: Estimation of cognitive damage from lead exposure 11

Step 3: Estimation of economic costs of cognitive damage 11

Step 4: Sensitivity analysis 12

Supplementary figures 14

Fig. S1 USA timeline of gasoline lead emission, preventive lead poisoning policies and BLL 14

Fig. S2 Global timeline of gasoline lead emission 15

Fig. S3 Schematic figure depicting the global lead cycle across the atmospheric, terrestrial and marine environments 16

Fig. S4 A collection of lead concentration profiles in soils around the world 17

Fig. S5 A collection of atmospheric lead deposition records derived from the analysis of ice cores 20

Fig. S6 The temporal variability of aerosol lead concentrations 21

Fig. S7 Quality control of estimated gasoline emitted lead from this study comparing with other estimated data from the literature or field measurements 22

Supplementary tables 24

Table S1 Estimates of gasoline lead emission in kilo metric tons per year in selected countries around the world/. Empty cells denote unavailability of data. 24

Table S2 Country name, temporal coverage, and sources used for existing gasoline lead emission data 29

Table S3 Country, temporal coverage and method used for newly estimated data in this study 30

References for supplementary information 31

Supplementary text

Text S1. History of leaded gasoline

With the first gallon of leaded gasoline sold in Dayton, Ohio, on February 2nd, 1923[1], humanity began a 100-year experiment to spread a dangerous toxin around the world. This commercial product powered transportation systems on land, air, and water as well as the mechanization of human labor. This changed the human culture. For nearly 50 years, the so-called "gift of God"[2] accounted for about 90% of the automotive fuel sold worldwide[3], a market dominance unlike any other commercial product. At the peak of its consumption in the 1960s and 1970s, more than 375,000 tons of lead went into the production of leaded gasoline globally – ranking lead among the top industrial chemicals in the world. The contribution of the production, distribution, and use of leaded gasoline to the global economy of its time seemed enormous – but increasing scientific evidence on the likely costs for public health and the environment were ignored (see timeline in Fig S2).

Lead is a naturally-occurring toxic metal used in a range of products that have been central industrialization (construction, plumbing, batteries, alloys, glass, paints, etc.)[4]. However, leaded gasoline was the first to be marketed worldwide in copious quantities, with its emission overwhelming its natural cycle in most environments. At the peak of leaded gasoline usage, anthropogenic emitted lead was responsible for >90% of atmospheric lead emissions[5] and >95% of lead in marine environments[6], and most of the lead came from automotive sources during the early to mid-20th century. The wanton release of fine-grained particles of lead[7] from millions of internal combustion engines spread with various natural processes to the most remote regions. Measurements of ice and surface waters from Greenland[4], Antarctic,[8] and the middle of the oceans[9,10] confirmed that no place on earth was free of lead contamination. Thus, emissions from leaded gasoline contaminated the world at a remarkable scale.

For much of the early part of the 20th century, the health impact from lead exposure went largely unnoticed because most doctors subscribed to the germ theory of disease causation. Since the common symptoms of many contagious diseases of the era were like those of symptomatic lead poisoning, cases were impossible to identify. Hence there is little information on how many children were poisoned in the early days of the leaded gasoline era. In the USA, for example, fewer than 100 children were treated in Children's Hospital of Boston for lead poisoning between 1924 and 1933[11]. As the number of cases increased with mass screening in subsequent decades, it quickly became obvious that symptomatic poisoning was only the tip of the iceberg with the much larger and submerged portion representing children with blood lead concentrations that were below the threshold associated with symptomatic poisoning (80 µg/dL at the time) but nevertheless were considered to be in excess of normal[12]. The worrisome group of children was said by public health officials to have a condition called "undue lead absorption"[12]. Subsequent large-scale surveys carried out in Chicago (1966) and New York (1970) confirmed that children with undue lead absorption (subclinical lead poisoning) numbered in the hundreds of thousands, and possibly millions[12].

The link between leaded gasoline, environmental lead exposure and the health impacts was drawn after viewing strong correlations between the sale of leaded gasoline, airborne lead levels, and blood-lead levels (Fig. S1). The Second National Health and Nutrition Examination Survey (NHANES II) showed a highly significant correlation between blood lead change, adjusted for demographic variables, and sale of leaded gasoline for children aged 6 months to 5 years[13]. BLL reductions in humans from cities and countries across five continents correlated strongly with reductions in the mean concentrations of lead in gasoline[14]. Such exposure/outcome relationships called into question that lead

paint and diet were dominant sources of lead exposure during the period of leaded gasoline usage[15]. The brief time lag between the sale of unleaded gasoline and the fall in BLL is inconsistent with the flow time for automotive lead from tailpipe through the soil and crops to human consumer which typically takes years (Fig. S1). This brief time lag ruled out diet as a dominant exposure pathway for automotive lead. Indeed, during the NHANES II study between 1976 and 1980, the estimated lead intake through diet showed no change in some segments of the population. Studies in many countries showed that in locations where lead was completely removed from gasoline, mean BLL fell to about 3 μg/dL or lower (Fig. S1, Fig. 2a) irrespective of the presence of other lead exposure pathways. The evidence was strong that the main exposure route to automotive lead was via inhalation and intake of lead contaminated (automotive) dusts by hand-to-mouth transfer for that period. It is apparent that while many children were exposed to lead from house paint and tap water, the global lead exposure was primarily the outcome of exposure to automotive lead exhausts.

By the early 1970s, three lines of evidence had emerged that changed the debate on policies and programs for protection of children from lead in their environment and presaged the removal of lead from gasoline. First, since the effects of lead poisoning are irreversible, screening children for high blood lead to qualify them for intervention programs came to be viewed as unethical and unacceptable practice[16]. Second, environmental economics provided robust models of cost–benefit analysis that called in serious doubt the presumed higher economic benefits of leaded gasoline in relation to health costs[17]. One study, for instance, showed that the benefit of reducing BLLs by 1 μg/dL in the USA population in 1986 was worth more than 17 billion dollars per year[18], compared with the estimated cost of $500 million for taking lead out of gasoline[19]. Third, reports showing that no level of lead in a child's blood could be considered as safe helped to sway public opinion and government policies towards the elimination of sources of lead as the best option for ending the lead exposure[20-22].

The introduction of unleaded gasoline and subsequent stringent policies to limit lead exposure in Europe and North America since 1970s resulted in international shift of environmental lead exposure. First, the lead industry was forced to step up its efforts to market the tetraethyl lead (TEL) additives in less developed countries (see timeline in Fig. S2)[23]. Second, impetus was given to exporting not only unwanted finished products from leaded gasoline to old and road-unworthy automobiles that burned leaded gasoline, but also dangerous lead processing industries from mining to recycling of lead acid batteries[24] to low-and-middle- income countries (LMICs)[25]. The international shift of environmental lead risks from the developed (outsourcing) to the developing (insourcing) countries delayed the adoption of lead-free gasoline in most developing countries (see the bottom timeline in Fig. S2). The lead exposure did not exactly end but instead was transitioned to the low- and middle-income countries. After the debut of lead-free gasoline, leaded gasoline was not completely phased out for about 24 years in the USA, 28 years in the European Union, 35 years in Asia Pacific and 49 years in Africa[26] (Fig. S2, Table S1).

Text S2. Lead in the atmospheric, marine, and terrestrial environment during the leaded gasoline period

The leakage of gasoline lead into the natural environment and its time-dependent distribution within and between atmospheric, marine, and terrestrial reservoirs has been studied since the early 1960s[27,28]. Elevated lead concentrations in Greenland ice cores and remote seawaters led scientists to conclude as early as 1963 that gasoline emissions have reached every corner of the globe[27]. These initial studies were helped by the fact that the isotopic compositions of gasoline lead used in Europe and North America were distinct from each other and from natural lead or lead contained in other pollutant sources such as coal burning, paints, etc.[27] The release of gasoline lead represented an inadvertent tracer experiment.

Atmospheric environment

Automotive emissions from the combustion of gasoline contains mainly fine particles consisting of soluble halides, e.g., lead chloride and bromide[7]. Because of small particle size, these lead halides are easily inhaled[29] and the lead quickly transfers to the blood stream due to its high solubility. Following the phasing out of leaded gasoline, coal combustion[5,30], aviation fuel (avgas)[31], along with the production, use, and recycling of lead-containing products[43,44] became the new dominant atmospheric emission sources. Lead released from automotive transportation makes today only a minimal contribution to the atmospheric lead, mainly through abrasion of brakes and other road furniture such as road markings[32]. Measurements in the world's major cities have shown that lead concentrations in aerosols have stabilized over the recent decade to as low as 10–50 ng/m^3 compared with >1000 ng/m^3 during leaded gasoline era (Fig. S6), but they are still hundreds of times higher than the anticipated natural aerosol composition[33-35]. This is associated with the resuspension of surface soils and new emission sources[36]. Mass balance calculations and isotope signatures demonstrate that much of the atmospheric lead today still arises from historical automotive emissions[33,37-39], for example about 30% to 40% in London's aerosols[33] today, suggesting that exposure to historical gasoline lead emissions is likely to continue. Lead-containing aerosols remain in the atmosphere up to 10 days[40] and are transported over thousands of kilometers into remote areas following atmospheric circulation patterns[4] (Fig. S2). Long-range atmospheric transport leads to significant transboundary lead pollution that can be traced quantitatively using lead isotopes[41-43]. For example, high-temporal resolution aerosol sampling combined with stable lead isotopes and concentration measurements, synoptic data, and atmospheric modeling demonstrated that lead detected in the atmosphere of Jerusalem, Israel, contained significant contributions of lead emitted in other countries[41]. Long-range transboundary pollution from China to the USA was initially proposed by comparing lead isotope ratios measurements of US west coast aerosols and Chinese coal, and confirmed by lead isotopes signatures determined in Western Pacific surface seawater[44,45] and Alaskan snow[43]. Transboundary pollution is now widely recognized as a critical environmental policy issue at regional[46] and global scale[47] and has been reported in almost every continent and ocean.

Marine environment

At the peak of leaded gasoline emissions in the 1970s, North Atlantic surface water downwind of the USA had the world's highest dissolved lead concentration, with levels of more than 150 pmol/kg (Fig. S3). These high concentrations decreased following the phasing out of leaded gasoline first in USA and then in Europe[48] (Fig. S2), and returned to the background concentrations with naturally sourced lead in some areas of the North Atlantic after 2010s[49]. The rapid removal of lead from the surface waters in the open ocean is due to the short residence time[50] (approximately 2 years in surface oceans, Fig. S3). For this reason, coral-based reconstructed lead concentrations in the North Atlantic Ocean surface water

closely track gasoline lead emissions from the USA and Europe[9]. In the Indian Ocean, emissions from both leaded gasoline and coal likewise track emissions from neighboring countries[30]. The lead that entered North Atlantic surface water in the 1970s and 1980s has been transported downwards by ocean currents and sinking particles. As a result, elevated concentrations of lead can now be found in seawater at greater depths between 1000 and 3000 m in the North Atlantic[10] (Fig. S3). Since the turn of the 21st century, the highest lead concentrations in open-ocean seawater are found in the surface waters of the north-western Pacific (60–100 pmol/kg)[44,45], and northern Indian Ocean (60 to 80 pmol/kg)[51,52], coinciding with contemporary high emission centers. In contrast, significantly lower concentrations have been measured in the Atlantic Ocean (20 to 40 pmol/kg)[10,49,53,54], Southern Ocean (5 to 25 pmol/kg)[55,56], and the Arctic Ocean (1 to12 pmol/kg)[57]. The estimated natural background level of lead in ocean surface water is 2 to 10 pmol/kg[9,30,58]. While atmospheric deposition is the main source of dissolved lead in the open ocean, coastal areas also receive lead input from river influx and surface runoff containing eroded topsoil enriched with legacy lead. The lead is subsequently removed from surface waters following adsorption on sinking particles, a process called scavenging (see Fig. S3). This process leads to the surprisingly low concentrations in river mouths near major human agglomeration centers, e.g., 42 to 84 pmol/kg at Yangtze River mouth[59] and ~150 pmol/kg at Ganges River mouth[60]. Some of these particles are subsequently transported seawards and release lead back into seawater[61]. These boundary processes (Fig. S3) generate a continuous release of lead into seawater in many marginal seas and continental slopes[45,61,62], governing lead concentrations in the marine environment for generations to come even after emissions have ceased.

Terrestrial environment

Enrichment of lead has been observed in top sections of urban and rural soils, lake sediments and ice cores around the world (Fig. S3, Fig. S4, Fig. S5)[63]. Isotope signatures links them largely to the deposition of atmospheric particulate matter derived from automotive emissions, especially close to highways and in urban soils near streets, but in remote areas also from mineral dust from remote areas[64] (see for example Fig. S4, Mt Cameroon). In the residential settings, besides atmospheric sources, runoff from residential house foundations and legacy paint are common lead sources[65]. The residence time of lead in soils and sediments is significantly greater than that observed in the marine and atmospheric environment. Lead migration and retention are controlled by dissolution/precipitation and adsorption/desorption reactions under changing pH and redox conditions, and field observations suggest limited mobility, likely as colloids, in most soil types (Fig. S3). Migration rates have been determined using a wide range of approaches. Using lead isotopes, contrasting soil profiles in Israel and Spain (temperate and arid) both showed slow migration rates ranging between 1 and 50 cm/century[66,67]. These migration rates suggest that gasoline-lead will remain in the topsoil for another 100 to 200 years, and that downward movements are slow with a retardation factor specific to the soil.

The restricted mobility most likely explains why to date groundwater contamination with lead is rare. Plant lead uptake and translocation into above-ground plant parts is usually very low as the exchangeable or acid extractable fraction of gasoline lead in soils is small[66,68-70]. However, consumption of lead-contaminated crops, where soil-splash particle contamination is likely, poses significant health risks[71].

Text S3. Methodologies in compiling and estimating the gasoline lead emission

Gasoline lead emission inventories used in this paper were either taken from literature compilations or established for countries where the data were unavailable. When different datasets overlapped, the mean of the estimated emission was taken. The complete dataset is plotted in Fig. S2 and summarized in Table S1, S2, and S3. Based on the gasoline usage data from the US Energy Information Administration[72], between 1980 and 2018, the gasoline usage from the countries included in this study comprise approximately 66 % of world's total usage. We expect our compilation and estimates to cover the world's major gasoline lead emission centers.

The method for estimating gasoline lead emissions used in this study followed methods applied in the literature[10,30,73]. Briefly, the emitted lead is calculated using the following equation:

Gasoline lead emission = (gasoline consumption) × (leaded gasoline market share) × (lead concentration in gasoline) × (emission factor)

, where the gasoline consumption is in liters per year (L/year) (if the source data are in barrels, a conversion of 1 barrel equals 159.5 L is applied), leaded gasoline market share is in percentage (summarized in Lee, et al. [30]), lead concentration in gasoline is in g/L, assumed to be the maximum allowable concentrations based on each countries' regulations at different points in time, emission factor is assumed to be 0.76, which is a typical value applied in a major country (e.g., 0.75 in Germany in 1990s[74], 0.76 in China in 2000s[30,75]).

The gasoline consumption datasets are from the US Energy Information Administration (https://www.eia.gov/petroleum/data.php)[72] and International Energy Agency (https://www.iea.org/)[76]. The data from the two sources are generally consistent for the same countries with the latter having more updated data coverage between 1990 and 2018.

Lead concentration in gasoline and leaded gasoline market share for countries in this study include:

India: Maximum allowable lead content in leaded gasoline before 1994 was 0.56 g/L[77]. In June 1994, gasoline with lower content (Pb = 0.15 g/L)[77] was introduced in Delhi, Mumbai, Kolkata, and Chennai. Unleaded gasoline (Pb = 0.013 g/L)[77] was introduced in 1995, and reached 100% market share by April 2000[77].

Indonesia: Maximum allowable lead content in leaded gasoline before 1989 was 0.7 g/L[78]. Between 1989 and 1999, the maximum allowable Pb in gasoline was 0.3 g/L[79]. Between 2000 and 2006, gasoline with lower lead content (Pb = 0.15g/L) were introduced[79], together with unleaded gasoline (Pb = 0.016 g/L) started to use in several major cities including Jakarta, Bali, Batam, and central Cirebon with unknown market share[80,81]. The whole country switched to unleaded gasoline in 2006[26].

Thailand: Lead content in gasoline was 0.84 g/L before 1983[79], decreased to 0.45 g/L between 1984 and 1988, further decreased to 0.4 g/L between 1989 and 1991, then to 0.15 g/L between 1991 and 1995[82]. Unleaded gasoline with lead content 0.013 g/L were used from 1996 onwards[79].

Vietnam: Before 1995, the lead content in the gasoline is not well documented. We therefore chose 0.84 g/L based on the general lead content in South East Asian countries in the 1980s[79]. Started in 1995, Vietnam decreased lead content to 0.4 g/L[83]. The country switched to unleaded gasoline (Pb = 0.013g/L) since July 1, 2000[84,85].

Philippines: Lead content in gasoline was 0.6 g/L before 1993[86]; and was reduced to 0.15 g/L between 1993 and December 23, 1999, where leaded gasoline replaced by unleaded gasoline (Pb = 0.013 g/L)[87]. Since 2005, Philippines further reduced the allowable Pb content to 0.005 g/L[88].

Brazil: Lead content in gasoline was 0.15 g/L before 1984[89], and decreased to 0.09 g/L in 1983[89], and further decreased to 0.06 g/L in 1987[89], and 0.026 g/L in 1989[1]. Leaded gasoline was completely eliminated in 1991[89].

The differences in gasoline consumption from US Energy Information Administration (https://www.eia.gov/petroleum/data.php)[72] and International Energy Agency (https://www.iea.org/)[76] range from 3% for Malaysia to up to *12%* for China, but are generally within 10%. We take *12%* as the worst-case uncertainty. Values chosen in estimating the uncertainty are underscored in bold italic fonts.

Information on leaded gasoline market share remains sparse. When the market share was unavailable, the estimates were based on the assumption of 100% leaded gasoline in the market, which is reasonable for some Asian countries as they applied an 'overnight-phase out strategy' with strong government enforcement and dominant gasoline supplier[79], but may cause significant over-estimation in European countries as the phase out of leaded gasoline was gradual[1]. Over year 1990-1991, large differences in the gasoline lead emission are observed among the data in Nriagu (1990), in Boyle et al (2014)[1,10], and in EU emission inventory report covering 1990–2020[90], likely due to the differences in assumed leaded gasoline market share. While we could not distinguish a more trustable estimate among the three studies, we find that difference in lead emission among three sources range from 806 tons/year in Germany in 1991 to 5516 tons/year in France in 1990, which equals to *20–30 %* of the peak gasoline lead emission of each country, respectively. The uncertainty due to non-available leaded gasoline market share leaded gasoline market share cannot be eliminated. Fortunately, this uncertainty is mainly relevant for a brief period only when the leaded gasoline usage use was rapidly falling, particularly in European countries. The lead emission from European countries between 1990 and 2000 should to this end be interpreted qualitatively and with caution.

Uncertainties arise by assuming lead concentration in gasoline as the maximum allowable lead concentration in gasoline set by each country, as the maximum allowable lead concentration in gasoline and the actual lead concentration may not be the same. However, we consider the maximum allowable lead content as firm as they are generally based on credible governmental reports.

Lastly, while we assume that the emission factor to be consistent with previous studies, we are aware that these may change across different automobile designs. Important to note is that despite the assumption that 76% of the gasoline lead is emitted immediately into the atmosphere, all the gasoline lead (100%) enters the environment eventually, as the remaining 24% of lead could deposit in the automobile exhaust systems and subsequent lost mechanically during driving and servicing[7]. Therefore, the ultimate emission of automobile Pb is likely between 76% and 100%, with the mean uncertainty assumed to be *12%*.

The propagated uncertainty in the final lead emission estimate is calculated using the sum of squares of the uncertainties discussed above, as the four variables in Eqn. 3 are independent from each other. The worst-case scenario happens when leaded and unleaded gasoline coexist in the market. When this happens, the maximum possible uncertainty is estimated to be 34% (the choices of values are underscored in bold italic fonts in Text S3). In other periods when there is either 100% leaded or 100% unleaded gasoline is in the market, the uncertainty is estimated to be 17% (the choices of values are underscored in bold italic fonts). This uncertainty can be reflected on the comparisons with the temporal

variability of the gasoline lead emission with paleo-environmental records (the comparison of emission with Greenland ice core record in Fig. S3 or comparing the estimated emission with direct measurements of lead in aerosol in Fig. S7. For example, the estimated gasoline lead emission from Singapore could explain 84% of the variability of the lead in Singapore aerosols (Fig. S7).

The temporal variability of the gasoline lead emissions included in this study compares well with various environmental data such as lead concentration in aerosol and paleo-environmental records. For example, the estimated gasoline lead emission from USA almost mirrors the ambient air lead concentration in the USA[1]. A similarly robust comparison also applies to Singapore aerosol lead concentration and is shown in Fig. S7. Lastly, the temporal variability of lead in corals from the middle of the ocean also seem to follow the lead emission data included in this study. These comparisons can be found in Kelly et al. (2009) for the North Atlantic Ocean[9] and Lee et al. (2014) for the Indian Ocean[30].

Text S4. Methodologies in estimating lead exposure

We detail here the methodology used to estimate the global economic costs of cognitive damage arising from childhood lead exposure presented in Fig. 3 and Table 1. All calculations were conducted using the R statistical programming language. Full replication materials are available from the GitHub repository for this paper at https://github.com/fditraglia/lead-review-maps.

Overview

Following is an overview of the methodology used to estimate the global economic costs of cognitive damage arising from childhood lead exposure, described in more detail below.

Step1: Approximate the distribution of blood lead levels (BLLs) among 0–19-year-olds in each country in 2019, using data from the Global Burden of Disease (GBD)[91].

Step 2: Estimate the average cognitive damage from childhood lead exposure in each country: convert the distribution of BLLs in each country to a mean number of IQ points lost per person, following Crump et al (2013)[92].

Step 3: Estimate the economic costs of cognitive damage: combine cross-country data on the returns to education–a proxy for the relative returns to cognitive skills–with estimates of the reduction in lifetime earnings from lower cognitive ability taken from the US Environmental Protection Agency.

Step 4: Conduct a sensitivity analysis of steps 1 and 2 by varying assumptions, methods, and input data.

Step 1: Approximation of the distribution of blood lead levels

This step relies on country-specific projections of lead exposure for individuals aged 0-19. These input data, taken verbatim from the 2019 GBD, are projections constructed via spatio-temporal Gaussian process smoothing using information collated from published reports of BLLs and a small number of blood lead surveys. For countries with little or no BLL data, the 2019 GBD imputes values based on a linear regression model that combines socio-economic data with information on the number of vehicles per capita and each country's history of leaded gasoline. Full details of the GBD methodology can be found on pages 139–144 of Appendix 1: Methods Appendix to "The Unfulfilled promise of prevention: the global burden of 87 risk factors, 1990–2019. A systematic analysis for the Global Burden of Disease Study 2019"[93].

We rely on two key variables from the GBD: average blood lead level (BLL) in micrograms per deciliter (µg/dL), the estimated number of individuals with a BLL >5 µg/dL, and the estimated number of individuals with a BLL >10 µg/dL. For the latter two summary statistics, the GBD provides separate counts for males and female individuals. We sum these to produce an overall count, and convert the counts to proportions using population data from the World Bank Open Data database[94]. In a small number of instances, the estimated count of individuals with a BLL >10 µg/dL is reported as "<1". To be conservative, any such values are replaced with zeros.

The mean BLL and fractions of individuals with BLL >5 and >10 µg/dL taken from the 2019 GBD are used to approximate the overall distribution of BLL in each country. The results presented in the body of the paper consider three different approximations. The "baseline" approximation chooses parameters of a Beta distribution supported on the interval [0, 100] for each country to exactly match the mean BLL value from the 2019 GBD and minimize the sum of squared vertical deviations between the fitted cumulative distribution function (CDF) and the fraction of individuals with BLL >5 µg/dL and those >10 µg/dL taken from the GBD. This approximation rules out BLL >100 µg/dL. Two

robustness checks reported in Table 1 use alternative distributional approximations. These are detailed below under “Step 4 - Sensitivity Analysis”.

Step 2: Estimation of cognitive damage from lead exposure

This step calculates the mean number of IQ points lost, per person aged 0–19 years, from lead exposure in each country. This calculation combines the estimated BLL distribution from each country, constructed in Step 1, with a function taken from row 9 of Table 5 from Crump et al. (2013) that relates lifetime mean BLL in early childhood to lost IQ points[92], namely $\beta \times \ln(BLL + 1)$ for $\beta=3.246$. This relationship is estimated from a linear regression of IQ points on ln(BLL + 1), “adjusted for site, HOME score, birth weight, maternal IQ, maternal education, maternal alcohol, maternal tobacco usage, and birth order”[92]. The measure of BLL used in the regression is the mean level “from 6 months to the time of the IQ test”[92]. We then compute the integral of $\beta \times \ln(BLL + 1)$ multiplied by the country-specific Beta distribution supported on [0, 100] from Step 1 to estimate the mean number of IQ points lost in that country, per person. A robustness check, described below in “Step 4 - Sensitivity Analysis” replaces the point estimate of β from Crump et al (2013) with the upper and lower endpoints of a 95% confidence interval[92].

Step 3: Estimation of economic costs of cognitive damage

This step translates the mean IQ points lost per person aged 0–19 years, calculated in Step 2 above, into economic terms. Following Klemick, Mason, and Sullivan (2020) the calculation begins with a value of 2.6% lower lifetime earnings (present value) for each 1 μg/dl of lead in a child’s blood[95]. This is a mean value across male and female individuals taken from the US Environmental Protection Agency (EPA), 2019 “Economic analysis of the final rule to revise the TSCA dust-lead hazard standards.”

Because the 2.6% value is computed from US data, however, it may not be directly applicable to countries with different returns to cognitive skills. To account for this possibility, country-specific figures are computed according to 0.026×Returns(X)/Returns(US), where Returns(X) is an estimate of the returns to education in country X and Returns(US) is the corresponding value for the US. This adjustment uses the returns to education as a proxy for the returns to cognitive skills. In countries with higher returns to education than the US, the estimated economic cost of lost IQ points is scaled up; in countries with lower returns to education, it is scaled down.

The relative returns to schooling used in this adjustment are taken from Montenegro and Patrinos (2021) and based on Mincer regressions[96], that is regressions explaining wages as a function of years of schooling and experience in the labor force[97]. Where possible, the returns to education used in the adjustment are from the year 2019. For countries with multiple years of returns to education but no information for 2019, a mean value for all years after 1990 is used instead. For countries with a single year of returns to education, this value is used regardless of year. For countries with no returns to education, a value is imputed based on the other countries in the same continent.

The results from Table 1 report the economic costs of cognitive damage from childhood lead exposure in two ways. The first, which we call “relative IQ cost” gives the mean foregone lifetime earnings in percentage points for a given country. A value of 5%, for example, would indicate that the average person aged 0–19 years in a particular country will earn 5% less over their lifetime because of lead exposure. The second, which we call “total IQ cost” converts these percentages into dollar values by multiplying them by GDP per capita at purchasing power parity in constant 2021 international dollars along with the population aged 0–19 years in 2019, using data from the World Bank, and summing across all countries in our dataset. Where available, GDP per capita is for the year 2019. Where

unavailable, the most recent value is used instead. Total IQ cost gives a sense of the order of magnitude of annual global income losses from lead exposure among 0–19 year olds. This calculation assumes that lifetime earnings losses accrue approximately proportionally each year, e.g., that a 5% lifetime earnings loss translates to approximately 5% lower income per year and uses each country's 2019 GDP/capita as a proxy for 1 year of individual income for the cohort of 0–19-year-olds in 2019. Both assumptions can only be expected to hold approximately: earnings losses may vary across an individual's working life, and the current cohort of 0–19-year-olds would be expected to face higher earnings when they enter the labor force than 2019 GDP/capita suggests due to economic growth.

Step 4: Sensitivity analysis

This step explores the sensitivity of the results computed following the methodology of steps 1–3 from above to changes in assumptions, methods, and input data.

Sensitivity to changes in the distributional approximation

The baseline approach described in Step 1 above uses a Beta distribution supported on [0, 100] to approximate the distribution of BLLs in each country based on summary statistics from the 2019 GBD. The results in Table 1 present two alternative approximations to explore the robustness of the baseline approach. The first alternative uses a lognormal distribution–a common choice for modeling BLLs–rather than a beta distribution but is otherwise identical to the baseline approach. The second alternative uses a three-point discrete distribution that provides a lower bound for BLLs in each country. This "lower bound approach" assigns a BLL of exactly 10 μg/dL to any individual with a BLL >10 μg/dL, a BLL of exactly 5 μg/dL to any individual with a BLL between 5 and 10 μg/dL, and a BLL of 0 to all other individuals. The lower bound approach sidesteps questions of how well the lognormal and beta distributions approximate the underlying unknown distribution of BLLs in each country, by taking a conservative approach that underestimates the BLL of every individual. Because all BLLs of 5 μg/dL and below are set to zero, this sensitivity analysis also sidesteps the challenge of accurately estimating the effect of low BLLs on IQ. Under the lower bound approach, only two values of the function $\beta\times\ln(BLL + 1)$ is used: $\beta\times\ln(6)$ and $\beta\times\ln(11)$. The lognormal and "lower bound" distribution approximations are then used to compute the average value of IQ points lost per person exactly as described in Step 2 above, taking the place of the beta distribution.

Sensitivity to changes in BLL to IQ function

The baseline approach described in Step 1 above computes IQ points lost as a function of BLL according to $\beta\times\ln(BLL + 1)$ with $\beta=3.246$, following row 9 of Table 5 from Crump et al (2013)[92]. This sensitivity analysis replaces the point estimate of 3.246 with the lower and upper endpoints of the associated 95% confidence interval reported in Crump et al. (2013)[92], namely [1.833, 4.659].

Sensitivity to changes in GBD input data

Information from the 2019 GBD is imperfect because many countries lack recent data on BLLs. (See footnote to Step 1 for more details.) This robustness check explores the sensitivity of the baseline calculations described above to the possibility that the GBD systematically underestimates the prevalence of elevated BLLs. It considers two counterfactuals that "scale up" the mean BLLs reported in the GBD, by 5% and 10%, respectively.

Note that Step 1 relies on both the mean BLL in each country and the fraction of individuals with BLL >5 and >10 μg/dL. In a counterfactual where the GBD is assumed to understate the prevalence of elevated BLLs, these fractions should also be adjusted upwards. The appropriate upwards adjustments

are determined by fitting two regression models, both using the “true” GBD input data. The first regression predicts the fraction of individuals with a BLL >5 µg/dL in each country using the mean BLL in that country. The second predicts the fraction of individuals with a BLL >10 µg/dL analogously. Both regressions are estimated using shape-constrained generalized additive models (GAMs) that ensure a monotonically increasing relationship. This prevents the estimated relationship between mean BLL and the fraction of individuals with BLLs above 5 µg/dL from declining slightly at extremely high values of mean BLL, where there are very few data. The fitted regressions are then used to construct counterfactual values of the fraction of individuals in each country with BLL >5 and >10 µg/dL in a thought experiment where mean BLLs are higher by a fixed fraction in all countries.

After the summary statistics from the GBD are adjusted upwards, Steps 2 and 3 are conducted as described above.

Supplementary figures

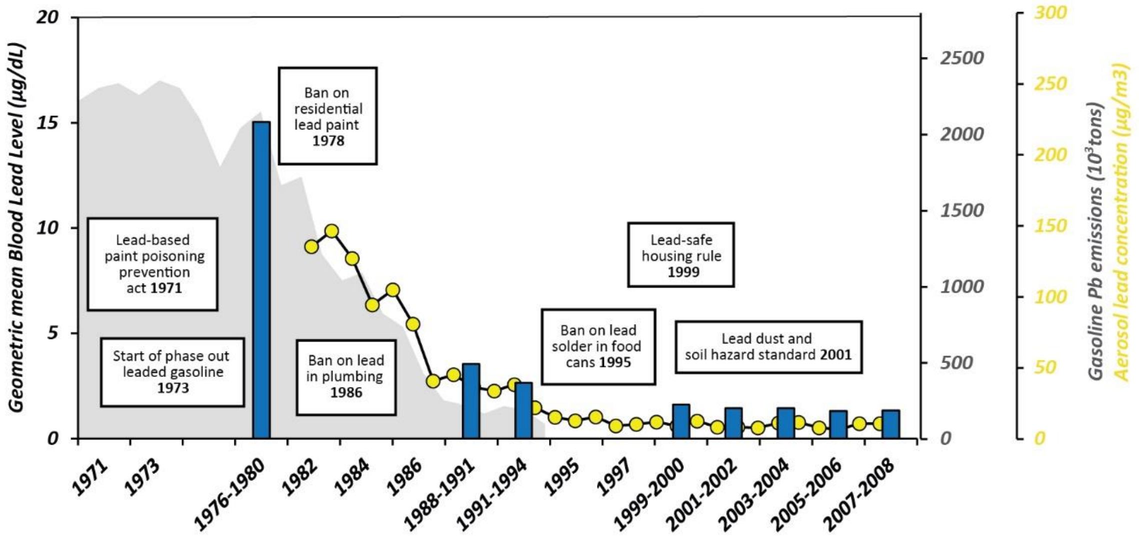

Fig. S1 USA timeline of gasoline lead emission, preventive lead poisoning policies and BLL

Timeline of gasoline lead emissions (grey shades) and preventative lead poisoning policies (white boxes), aerosols lead concentrations (yellow circles), and the geometric mean of blood lead levels (blue bars) in the USA. Lead emission data replotted from[1]. Blood lead level data taken from [13,98,99]. Aerosol lead content data taken from[61]. Summary of policies taken from Brown and Stephen (2012)[99].

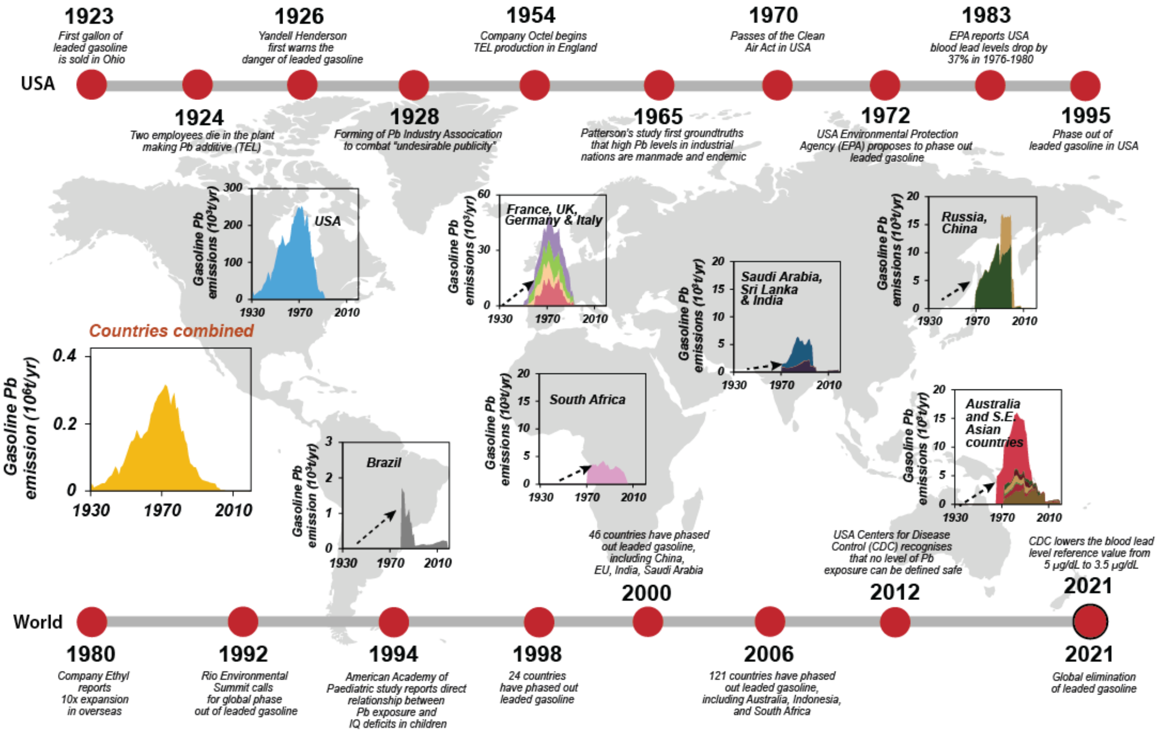

Fig. S2 Global timeline of gasoline lead emission

Estimated atmospheric lead emissions from gasoline from selected countries across the world with key events illustrated in the context of the 100 years' timeline of leaded gasoline in the USA (top) and the world (bottom)[100]. The countries presented in this figure comprise about 65 % of the global gasoline usage[76]. Arrows in the inset figures illustrate likely trends of gasoline lead emission where emission data are not available. For example, leaded gasoline was introduced in Japan in 1927, United Kingdom in 1928, Canada in 1926, Ireland and Australia in 1932, Italy in 1935, Germany in 1936, Mexico in 1937, France in 1939, and Russia in 1942[101]. Each of the inset figures is plotted as the integrated emission of the countries mentioned. Note the difference in scales among countries. The combined emission is illustrated in the leftmost inset figure in yellow. The total integrated emission is plotted in Fig. 1 and Fig S3 as yellow filled areas. Table S1 presents emission data compiled from the literature along with new estimates constructed for this study. Full methodological details appear in Text S3.

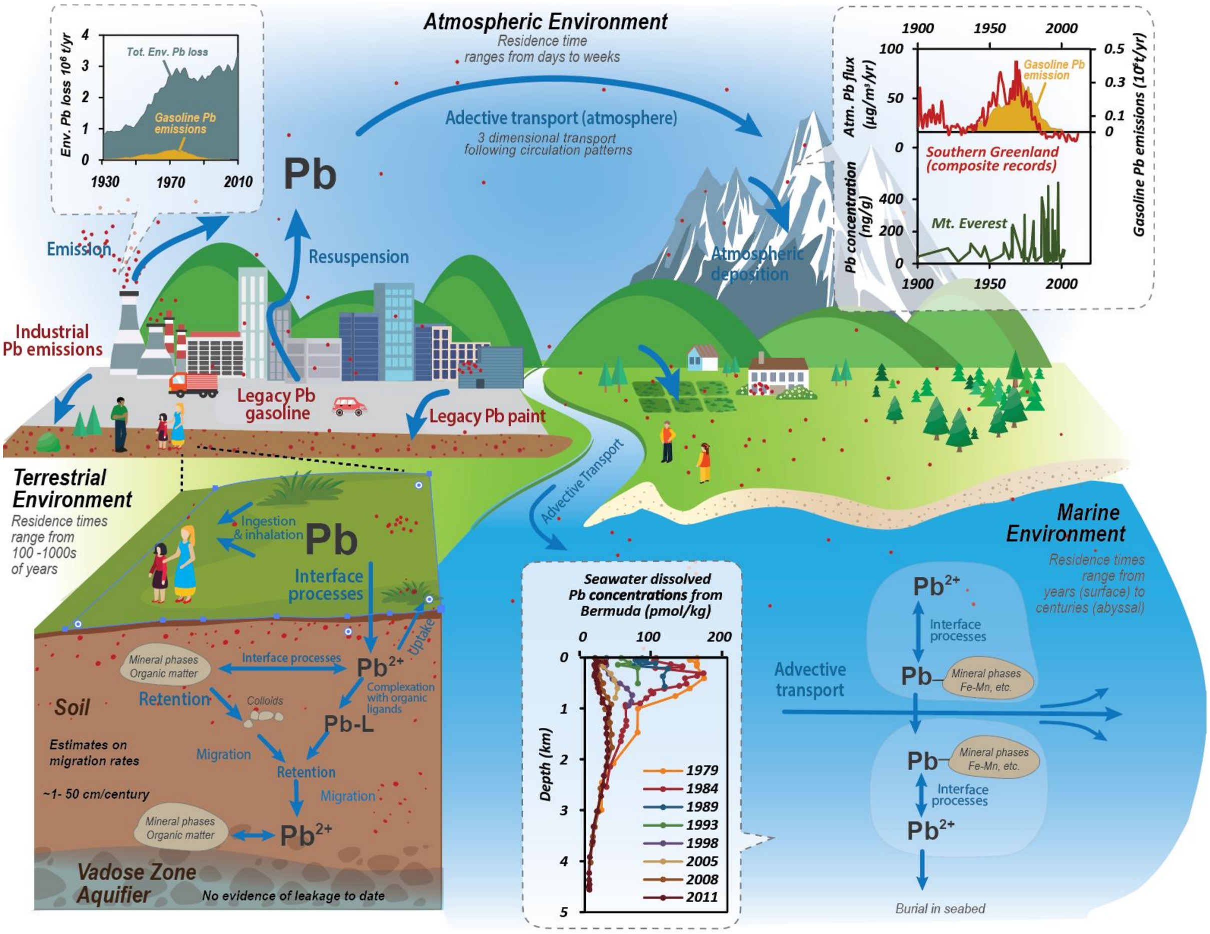

Fig. S3 Schematic figure depicting the global lead cycle across the atmospheric, terrestrial and marine environments

Schematic illustration of the global lead cycle and leakage into the atmospheric, terrestrial, and marine environments with major sources (red) and processes (blue) shown. The top left inset figure illustrates the time series of estimated gasoline lead emission (10^6 tons/year, yellow filled area, same as the combined total emission in Fig. S2) and the total leakage of lead (10^6 tons/year) occurring during mining, production, usage, recycling and disposal of lead-containing products into the environment[32]. The top right inset figures show records of atmospheric lead deposition derived from Greenland[4] ice cores and the reconstructed lead concentration in a Mount Everest ice core[102]. We show the latter due to its location near main contemporary emission center, despite suboptimal resolution. The Greenland record agrees well with the total integrated gasoline lead emissions (yellow filled area in 10^3 tons/year). Mount Everest record is located near the world's new emission centers in Asia and hence is showing an increasing lead concentration in recent decades. The bottom inset figure shows depth profiles of dissolved lead concentration in seawater collected at Bermuda (North Atlantic Ocean)[10] demonstrating decreasing concentration in the surface from 1979 to 2011, accompanied by a development of the subsurface maximum due to advective transport and scavenging.

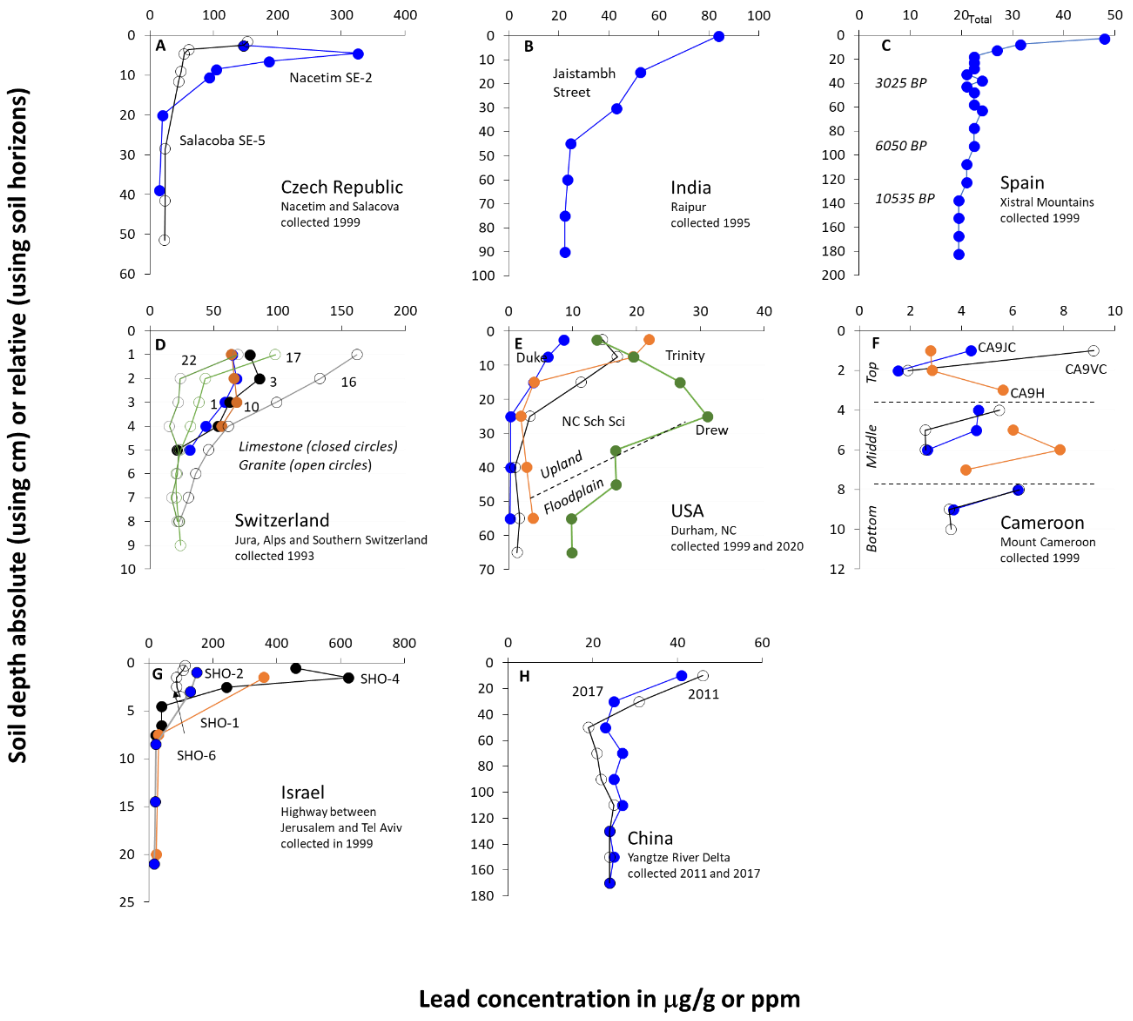

Fig. S4 A collection of lead concentration profiles in soils around the world

A collection of depth profiles for total lead (expressed in µg/g, mg/kg, or ppm) determined in various urban and rural soils at locations around the world. The profiles highlight the general trend of lead accumulation in the topsoil but also show evidence for mobility in certain soil types (panels E and H). Depth is indicated either absolute using metric measures, or relative using genetic soil horizons down to a C or B/C horizon (panels D and F). Each profile in panel F contains three separate sub soil profiles (top, middle, bottom) due to repeated burial of soil sequences following volcanic eruptions. The sources of lead in the topsoils are in general anthropogenic (i.e., gasoline, industrial, or paint lead) but there is also enrichment due to natural processes (panel F where mineral dust transported from the Sahara Desert is the main source for the lead in the upper parts of each section). It is evident that most of the anthropogenic lead in soils resides in the top 20 to 30 cm. The order of lead affinity to soil components may be summarized as Fe-oxides > organic matter > silicates. Panel A: Two soil depth profiles from forested sites (Nacetin, Salacova Lhota) in the Czech Republic[69] sampled in 1999. Nacetin in the Krusne Hory Mountains is a contaminated site, which has been severely polluted by emissions from heavy industry and coal-burning power stations. The sampling site is situated in a forested plot isolated from heavy road traffic. The Salacova Lhota area in the Bohemian-Moravian Uplands of the central Czech Republic has been subjected to significant lead contamination. Lead in the deep mineral soils is of natural origin and primarily associated with silicates and Fe-oxides. Natural lead associated with surface bound and organic matter fractions in mineral soils accounts for 7% to 15%. Anthropogenic lead is

concentrated primarily in the organic horizons and is associated with surface-bound and organic matter fractions in which the proportion of total lead is 33% to 50% and 23% to 47%, respectively. Panel B: One lead concentration depth profile of a soil along a busy street (Jaistambh) in Raipur, central India, collected in 1995. The central part of India is rich in natural resource materials, including ores of iron, aluminum, calcium and fossil fuel (coal) that are mined and processed. Steel plant, thermal power plant, and automobile exhaust were the main sources for lead emissions in the urban environment of this region during the study period. Panel C: One soil depth profile from a site in the Xistral Mountains of north-western Spain collected in 1999[66]. Sample were collected from a colluvial soil located formed over the last 10,000 years at a foot slope at 600 masl. A soil monolith referred as NDS measuring 185 cm in length was taken. The soil is organic-rich, acidic, and unsaturated in base cations but saturated with aluminum. Based on the history of soil formation, the lead migration rate was estimated at 0.01 cm/year. At this migration rate, lead would be retained in the soil column for ~20 kyrs. Shown for NDS are total concentrations data as well as calculated age depths. Panel D: Six soil depth profiles formed on different bedrocks (profiles Nos 2, 3 and 10 on limestone, Nos 16, 17 and 22 on granite) collected across Switzerland in 1993[103]. They were chosen from a systematic 8 × 8 km grid of 173 well characterized forest sites located in the Jura Mountains, in the Alps and in Southern Switzerland. Sample were collected from all genetic soil horizon down to a C or B/C horizon. Solubility of lead decreased with soil depth[103]. Panel E: Four soil depth profiles of total soil lead from sites across forested upland (Duke Park, NC School Sci Math, Trinity Watts Park) and floodplain (Drew Granby Park) parks or park-like areas not adjacent to streets and houses in Durham, North Carolina, USA[65]. The floodplain lead profile reflects the extent of human alterations meant to minimize flooding and promote sediment transport. Panel F: Three soil profiles (CA9JC, CA9VC and CA9H) sampled on the southeastern and the north easter parts of Mount Cameroon, Cameroon[64]. They are paleo soil successions developed from alteration of basaltic lava in the deepest units of the profiles. Lead isotope compositions and concentrations in the uppermost horizons were different from underlying horizons in the soil sequences. Anthropogenic contamination from leaded gasoline cannot explain the shifts observed, and a natural source must be evoked, likely Saharan dust delivered during winter when dirty warm Harmattan wind blows from the northeast. Panel G: Soil profiles collected from four sites 8 to 23 m away from a major highway between Jerusalem and Tel Aviv in Israel in 1999 (SHO-4 at 8 m, SHO-6 at 10 m, SHO-1 at 11 m and SHO-2 at 12 m distance)[68]. A selective sequential dissolution procedure was used to determine the distribution of lead between different soil components, i.e., soil carbonate, organic matter, Fe-oxides and hydrous oxides, and aluminosilicates. Natural lead was associated with aluminosilicates and Fe-oxides, and only a small fraction with soil carbonate and organic matter. The distribution of anthropogenic lead, which accumulates in the upper part of the soil profile, is ~40% with soil carbonates, ~10% with organic matter, ~35% with Fe-oxides, and only ~15% with aluminosilicates. Based on concentration and isotopic composition of total lead, the deeper horizon of the roadside soil (10–30 cm) and the soil sampled 500 m from the highway seem uncontaminated. However, the isotopic composition of the labile components in these unpolluted soils indicates that these soils contained anthropogenic lead. This contamination implies that over the period of vehicle pollution (~40 yr), a small fraction of the gasoline lead has penetrated through the entire roadside soil profile (25–30 cm). Panel H: Two paddy soil profiles collected at a cropland in the Mid Yangtze River Delta in 2011 and 2017[104]. Distinct lead concentration gradient in the soil indicates a substantial exogenous lead in the shallow horizons in both 2011 and 2017. There is evidence of lead vertical migration between 2011 and 2017. Compared with 2011, the concentrations of lead in the shallow soils in 2017 decrease while in the deep soils (40 to 120 cm) lead concentrations increase, reflecting the ongoing migration of lead from topsoil into the deeper soil.

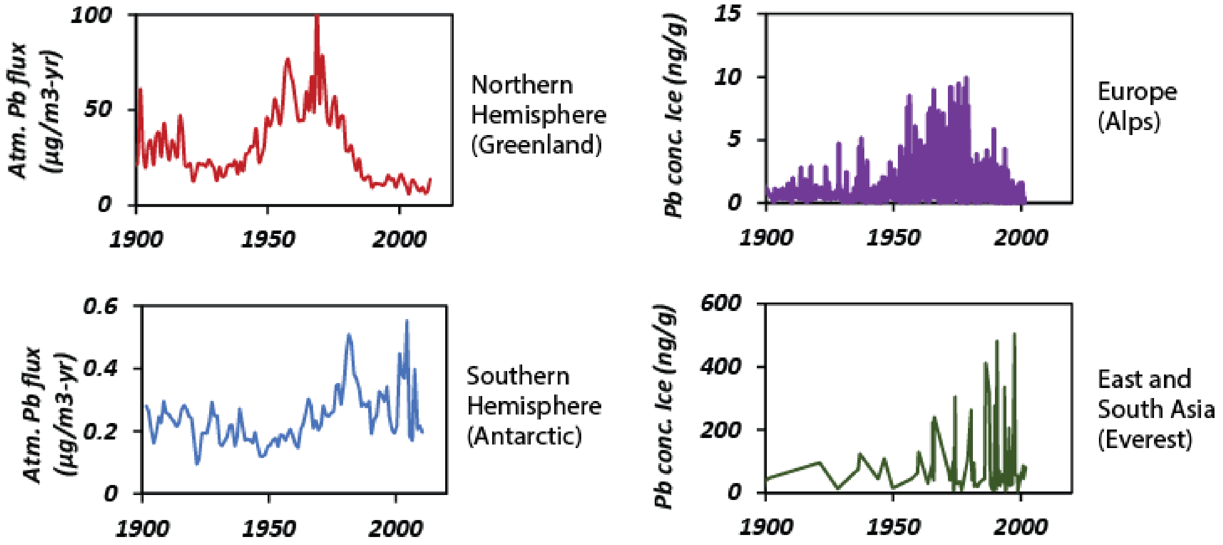

Fig. S5 A collection of atmospheric lead deposition records derived from the analysis of ice cores

A collection of ice core records from Greenland[4], Antarctica[105], Europe (Alps)[106], and Asia (Mt Everest)[102]. The Greenland and Antarctic records are composite records of atmospheric lead flux that are expected to reflect the hemispheric-scale lead deposition. While the records in the Alps and Mt Everest are lead concentrations in specific cores from Europe and Asia, the peak lead deposition in the northern hemisphere occurs earlier than southern hemisphere, and the latter has significant anthropogenic lead deposition today.

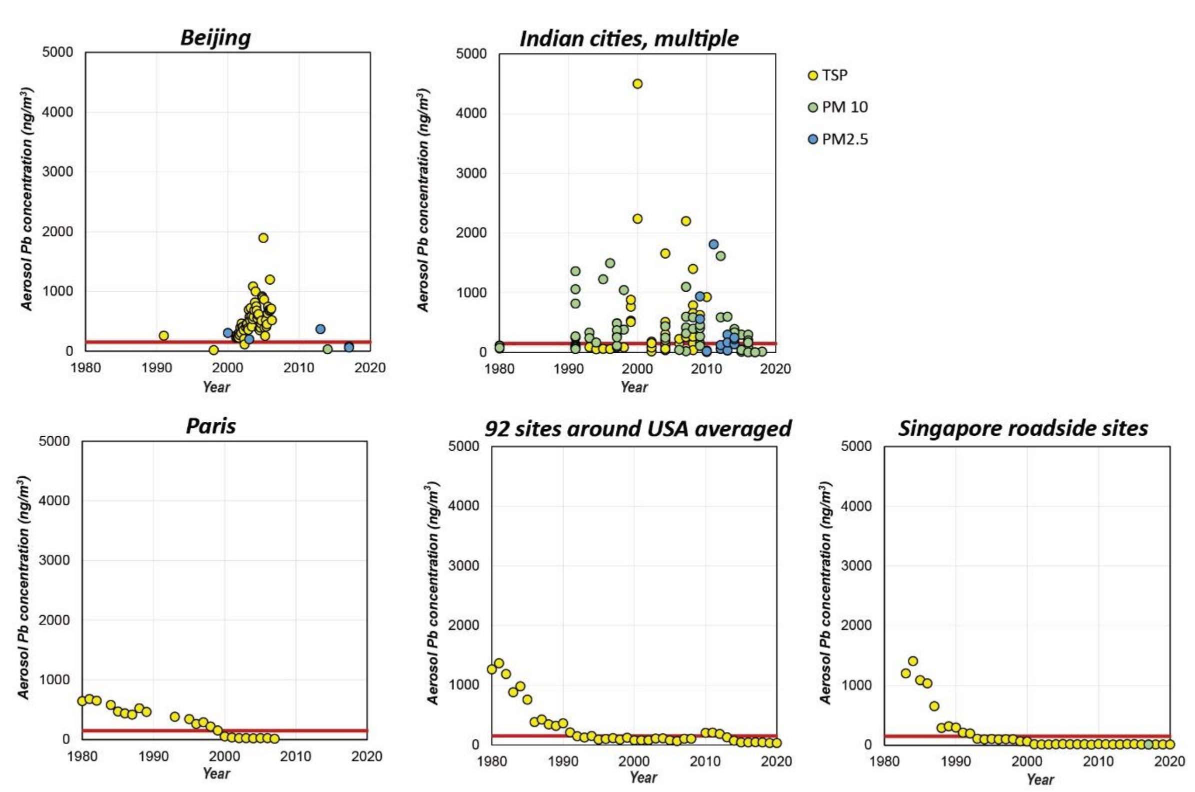

Fig. S6 The temporal variability of aerosol lead concentrations

Temporal variability of aerosol lead concentrations (ng/m^3) in selected cities around the world. Data from Paris, the USA, and Singapore are annual means of several stations as part of governmental monitoring programs; data from Beijing and Indian cities are combined datasets from discrete studies due to sparseness of data. The red horizontal line denotes the current US EPA air quality standard of 150 ng/m^3. Aerosol lead concentration data compiled from Beijing[107-115], Indian cities[116-162], Paris[163], USA[164], and Singapore[34,165-167]. The data associated with the figure are summarized at doi:10.25540/HXFN-68FM.

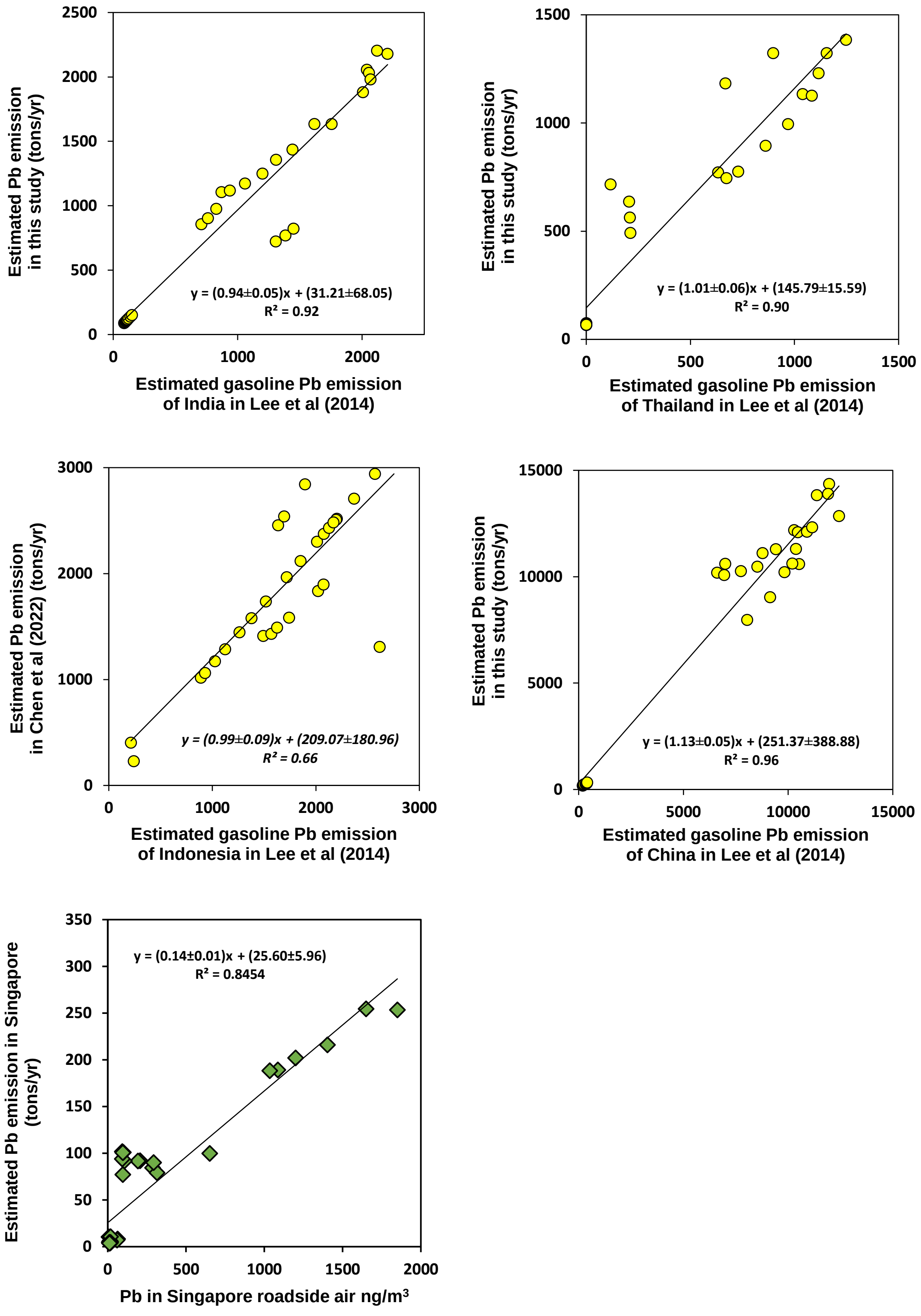

Fig. S7 Quality control of estimated gasoline emitted lead from this study comparing with other estimated data from the literature or field measurements

Comparions of estimated lead emitted from gasoline from various literature sources included in this study. Yellow circles are comparing with other studies, green diamonds are comparing with actual observations[165,166]. Sources of data are summarized in Table S2 and S3. The reason for the largely skewed Chinese emission data is that China phased out leaded gasoline within a few years[75,168,169], which appears in the annual resolution similar to an overnight phase out.

Supplementary tables

Table S1 Estimates of gasoline lead emission in kilo metric tons per year in selected countries around the world. Empty cells denote unavailability of data.

The editable data file is available at https://doi.org/10.25540/HXFN-68FM

Year	India	Sri<br>Lanka	Saudi<br>Arabia	Indon<br>esia	Malay<br>sia	Singa<br>pore	Thailand	Philip<br>pines	Vietnam	Austra<br>lia	South<br>Africa	Brazil	China	Russia	USA	UK	Italy	Germ<br>any	France
1930																			
1931															21.92				
1932															9.87				
1933															13.43				
1934															15.66				
1935															18.19				
1936															21.87				
1937															24.10				
1938															23.26				
1939															29.72				
1940															39.11				
1941															43.82				
1942															48.21				
1943															60.00				
1944															73.87				
1945															64.01				
1946															47.85				
1947															71.44				

Year	India	Sri Lanka	Saudi Arabia	Indonesia	Malaysia	Singapore	Thailand	Philippines	Vietnam	Australia	South Africa	Brazil	China	Russia	USA	UK	Italy	Germany	France
1948															76.98				
1949															87.61				
1950															104.08				
1951															118.09			0.71	2.00
1952															134.01			0.71	2.31
1953															146.30			0.83	2.76
1954															146.71			1.18	3.14
1955															152.74	4.45		1.44	3.18
1956															172.85	4.47		1.66	3.89
1957															158.68	4.67		2.47	4.11
1958															145.08	5.63		3.08	4.00
1959															145.54	5.53		3.76	4.35
1960															150.04	5.88	5.15	3.98	5.07
1961															153.56	5.62	5.61	4.56	6.21
1962															155.62	6.62	5.93	5.56	6.19
1963															174.85	8.07	5.52	6.24	6.27
1964															202.88	9.37	5.58	6.47	7.13
1965										4.87					204.97	9.31	7.07	7.36	7.49
1966										5.06					223.72	9.84	9.34	7.88	8.33
1967										5.34					225.59	9.80	13.95	8.58	8.39
1968										5.65					237.69	9.53	13.97	8.90	9.59
1969										6.04					246.87	7.95	11.09	9.24	10.79

Year	India	Sri Lanka	Saudi Arabia	Indonesia	Malaysia	Singapore	Thailand	Philippines	Vietnam	Australia	South Africa	Brazil	China	Russia	USA	UK	Italy	Germany	France
1970										6.40					250.24	9.75	11.88	10.54	10.62
1971	0.88	0.07	0.45	0.95	0.55	0.00	0.67		0.82	6.74	2.97		3.13		242.04	11.05	15.18	9.45	12.06
1972	0.92	0.08	0.51	1.00	0.58	0.00	0.68		0.84	7.13	3.18		3.65		252.45	11.88	14.49	9.04	12.98
1973	0.87	0.08	0.60	1.10	0.69	0.24	0.84		0.82	7.38	3.41		4.28		247.05	11.92	12.25	8.73	13.45
1974	0.73	0.06	0.74	1.20	0.74	0.24	0.95		0.67	7.26	3.21		5.16		225.11	11.46	10.49	8.73	13.42
1975	0.72	0.06	0.99	1.35	0.78	0.26	0.90		0.67	7.77	3.54		5.57		191.30	11.30	10.17	6.44	14.04
1976	0.73	0.06	1.31	1.48	0.88	0.26	0.85		0.22	8.31	3.55		5.61		218.99	11.60	9.18	3.45	14.03
1977	0.76	0.07	1.69	1.63	0.77	0.27	1.09		0.14	8.77	3.42		6.07		230.22	11.12	12.29	3.43	12.62
1978	0.82	0.08	2.14	1.84	0.82	0.27	1.33		0.16	9.15	3.57		6.69		178.51	10.94	12.10	3.63	12.80
1979	0.81	0.07	2.45	1.99	0.96	0.30	1.36		0.20	9.46	3.13		7.17		184.60	10.68	13.33	3.98	11.88
1980	0.90	0.08	3.24	2.16	1.16	0.32	1.32	0.94	0.27	9.42	3.26	1.71	8.81		129.95	9.83	16.55	3.85	11.79
1981	0.99	0.08	3.78	2.36	1.24	0.25	1.24	0.89	0.23	9.46	3.59	1.61	8.40		111.57	10.37	9.30	3.64	9.81
1982	1.03	0.08	4.15	2.35	1.34	0.25	1.17	0.88	0.24	9.72	3.73	1.58	8.52		116.94	9.43	8.73	3.73	9.59
1983	1.12	0.09	4.87	2.23	1.32	0.20	1.11	0.82	0.26	9.58	3.88	0.87	9.01		88.34	8.89	8.86	3.59	9.66
1984	1.22	0.10	5.03	2.28	1.21	0.22	0.70	0.70	0.25	9.80	4.18	0.92	9.50		78.89	8.44	5.41	3.09	10.23
1985	1.33	0.11	4.68	2.33	1.04	0.19	0.71	0.64	0.27	9.72	4.02	0.96	9.95		46.15	7.80	5.03	2.99	10.33
1986	1.44	0.11	3.92	2.54	0.84	0.19	0.75	0.66	0.28	9.66	3.51	1.10	10.36		27.35	4.33	3.39	2.46	11.05
1987	1.63	0.12	3.82	2.76	0.89	0.10	0.88	0.74	0.30	9.03	3.24	0.74	11.24		23.93	4.21	2.18	2.40	11.05
1988	1.69	0.14	3.83	2.75	0.93	0.08	0.98	0.80	0.43	8.71	3.50	0.73	12.61		17.87	3.42	1.51	2.43	10.75
1989	1.95	0.11	2.96	1.29	0.86	0.08	1.09	0.90	0.40	8.28	3.04	0.35	13.16		23.18	3.41	2.71	2.02	7.12
1990	2.05	0.07	2.73	1.44	0.43	0.09	1.10	0.92	0.56	7.83	2.57	0.41	12.91		20.15	3.04	0.75	0.72	2.80
1991	2.04	0.07	2.84	1.53	0.49	0.09	0.93	0.83	0.49	5.31	2.65	0.08	8.01		10.66	3.15	2.27	0.60	2.14

Year	India	Sri Lanka	Saudi Arabia	Indonesia	Malaysia	Singapore	Thailand	Philippines	Vietnam	Australia	South Africa	Brazil	China	Russia	USA	UK	Italy	Germany	France
1992	2.02	0.07	3.19	2.04	0.40	0.09	0.35	0.89	0.57	3.14	2.66	0.08	9.09	7.71		2.40	2.47	0.02	0.02
1993	2.19	0.07	3.51	2.12	0.38	0.09	0.39	0.25	0.76	2.23	2.76	0.09	10.56	6.84			0.03	0.02	0.02
1994	2.16	0.08	3.70	2.37	0.34	0.09	0.42	0.27	0.85	1.32	2.89	0.09	10.02	6.64			0.03	0.02	0.02
1995	1.02	0.04	3.66	2.61	0.27	0.10	0.42	0.30	0.40		3.28	0.10	10.41	6.63			0.02	0.02	0.02
1996	1.08	0.05	3.79	2.28	0.22	0.10	0.03	0.34	0.42		3.11	0.12	10.84	6.31			0.02	0.02	0.02
1997	1.14	0.05		2.55	0.18	0.10	0.04	0.36	0.49		3.00	0.12	11.28	6.36			0.02	0.02	0.02
1998	0.78	0.05		2.61	0.09	0.08	0.04	0.41	0.46		2.88	0.12	11.51	6.02			0.02	0.02	0.02
1999	0.83	0.04		1.96	0.08	0.01	0.03	0.41	0.54		2.72	0.12	11.74	5.50			0.02	0.01	0.01
2000	0.09	0.04		1.45	0.05	0.01	0.03	0.03	0.58		2.45	0.11	12.64	4.78			0.01	0.00	0.00
2001	0.09	0.03		1.50	0.05	0.01	0.03	0.04	0.64		2.27	0.10	0.19	5.11			0.01	0.00	0.00
2002	0.10	0.00		1.56	0.05	0.01	0.04	0.04	0.03		2.11	0.11	0.20	5.24			0.00	0.00	0.00
2003	0.11	0.00	0.00	1.66	0.06	0.01	0.04	0.04	0.03	0.07	1.55	0.11	0.22	0.13			0.00	0.00	0.00
2004	0.11	0.00	0.00	1.93	0.06	0.01	0.04	0.04	0.03	0.07	0.99	0.11	0.25	0.14			0.00	0.00	0.00
2005	0.12	0.00	0.00	1.99	0.06	0.01	0.04	0.01	0.04	0.08	0.33	0.12	0.29	0.14			0.00	0.00	0.00
2006	0.12	0.00	0.00	0.31	0.06	0.01	0.04	0.01	0.04	0.07	0.00	0.12	0.32	0.14			0.00	0.00	0.00
2007	0.14	0.00	0.00	0.23	0.07	0.01	0.04	0.01	0.04	0.07	0.00	0.13	0.35	0.15			0.00	0.00	0.00
2008	0.15	0.00	0.00	0.27	0.06	0.01	0.03	0.01	0.05	0.07	0.00	0.15	0.37	0.16			0.00	0.00	0.00
2009	0.17			0.29	0.14	0.01	0.07	0.01	0.05			0.16	0.33	0.15			0.00	0.00	0.00
2010	0.19			0.30	0.13	0.01	0.07	0.01	0.06			0.18	0.38	0.16			0.00	0.00	0.00
2011	0.20			0.34	0.12	0.00	0.07	0.01	0.06			0.18	0.42	0.17			0.00	0.00	0.00
2012	0.21			0.37	0.13	0.00	0.07	0.01	0.06			0.19	0.45	0.17			0.00	0.00	0.00
2013	0.23			0.38	0.19	0.00	0.08	0.02	0.06			0.21	0.51	0.18			0.00	0.00	0.00

Year	India	Sri Lanka	Saudi Arabia	Indonesia	Malaysia	Singapore	Thailand	Philippines	Vietnam	Australia	South Africa	Brazil	China	Russia	USA	UK	Italy	Germany	France
2014	0.25			0.40	0.19	0.00	0.08	0.02	0.06			0.22	0.54	0.19			0.00	0.00	0.00
2015	0.28			0.38	0.20	0.00	0.09	0.02	0.08			0.23	0.63	0.18			0.00	0.00	0.00
2016	0.32			0.40	0.20	0.00	0.10	0.02	0.08			0.22	0.67	0.18			0.00	0.00	0.00
2017	0.34			0.41	0.21	0.00	0.10	0.02	0.09			0.22	0.70	0.17			0.00	0.00	0.00
2018	0.38			0.42			0.09	0.02	0.09			0.22	0.66				0.00	0.00	0.00
2019																	0.00	0.00	0.00

Table S2 Country name, temporal coverage, and source used for existing gasoline lead emission data

Country	Temporal coverage	Source
Australia	1965-1994	Lee et al 2014[30]
Australia	2003-2008	Lee et al 2014[30]
China	1971-2008	Lee et al 2014[30]
France	1951-1991	Boyle et al 2014[10]
France	1990-2020	EU emission inventory report 1990-2020[90]
Germany	1951-1991	Boyle et al 2014[10]
Germany	1990-2020	EU emission inventory report 1990-2020[90]
India	1970-2008	Lee et al 2014[30]
Indonesia	1971-2008	Lee et al 2014[30]
Indonesia	1971-2018	Chen et al 2022[73]
Italy	1951-1992	Boyle et al 2014[10]
Italy	1990-2019	EU emission inventory report 1990-2020[90]
Malaysia	1971-2008	Lee et al 2014[30]
Malaysia	1970-2017	Chen et al 2022[73]
Russia	1990-2002	Zurbrick et al 2017[44]
Saudi Arabia	1971-2008	Lee et al 2014[30]
Singapore	1971-2008	Lee et al 2014[30]
Singapore	1973-2017	Chen et al 2022[73]
South Africa	1971-2008	Lee et al 2014[30]
Sri Lanka	1971-2008	Lee et al 2014[30]
Thailand	1971-2008	Lee et al 2014[30]
United Kingdom	1955-1992	Boyle et al 2014[10]
United States	1931-1991	Nriagu 1990[1], Boyle et al 2014[10]

Table S3 Country, temporal coverage and method used for newly estimated data in this study

Country	Temporal coverage	Method used for estimation
China	1980-2018	Follows Lee et al (2014)[30] and Chen et al (2022)[73] for consistency
India	1980-2018	Follows Lee et al (2014)[30] and Chen et al (2022)[73] for consistency
Vietnam	1970-2018	Follows Lee et al (2014)[30] and Chen et al (2022)[73] for consistency
Philippines	1980-2018	Follows Lee et al (2014)[30] and Chen et al (2022)[73] for consistency
Brazil	1980-2018	Follows Lee et al (2014)[30] and Chen et al (2022)[73] for consistency
Thailand	1980-2018	Follows Lee et al (2014)[30] and Chen et al (2022)[73] for consistency

References for supplementary information

1 Nriagu, J. O. The rise and fall of leaded gasoline. *Science of the total environment* **92**, 13-28 (1990).

2 US Public Health Service. Proceedings of a conference to determine whether or not there is a public health question in the manufacture, distribu-tion or use of tetraethyl lead gasoline. *Public Health Bulletin* **158**, 69-67 (1925).

3 National Research Council. *Lead; Airborne Lead in Perspective*. (Committee on Biologic Effects of Atmospheric Pollutants, National Academy Press, 1972).

4 McConnell, J. R. *et al.* Pervasive Arctic lead pollution suggests substantial growth in medieval silver production modulated by plague, climate, and conflict. *Proceedings of the National Academy of Sciences* **116**, 14910-14915 (2019).

5 Nriagu, J. O. Global inventory of natural and anthropogenic emissions of trace metals to the atmosphere. *Nature* **279**, 409-411 (1979).

6 Flegal, A. R. Lead in tropical marine systems: a review. *Science of the Total Environment* **58**, 1-8 (1986).

7 Seyferth, D. The Rise and Fall of Tetraethyllead. 2. **22**, 5154-5178 (2003).

8 Van De Velde, K. *et al.* Pb isotope record over one century in snow from Victoria Land, Antarctica. *Earth and Planetary Science Letters* **232**, 95-108 (2005).

9 Kelly, A. E., Reuer, M. K., Goodkin, N. F. & Boyle, E. A. Lead concentrations and isotopes in corals and water near Bermuda, 1780–2000. *Earth and Planetary Science Letters* **283**, 93-100 (2009).

10 Boyle, E. A. *et al.* Anthropogenic lead emissions in the ocean: The evolving global experiment. *Oceanography* **27**, 69-75 (2014).

11 Vogt, E. C. & Mckhann, C. F. Lead Poisoning in Infants and Children: Roentgenological Findings. *Radiology* **22**, 87-92 (1934).

12 English, P. C. *Old paint: A medical history of childhood lead-paint poisoning in the United States to 1980*. (Rutgers University Press, 2001).

13 Mahaffey, K. R., Annest, J. L., Roberts, J. & Murphy, R. S. National estimates of blood lead levels: United States, 1976–1980: association with selected demographic and socioeconomic factors. *New England Journal of Medicine* **307**, 573-579 (1982).

14 Thomas, V. M., Socolow, R. H., Fanelli, J. J. & Spiro, T. G. Effects of reducing lead in gasoline: an analysis of the international experience. *Environmental Science & Technology* **33**, 3942-3948 (1999).

15 US Environmental Protection Agency. Review of the national ambient air-quality standards for lead: Assessment of scientific and technical information. Report EPA-450/02-89/022 (Environmental Protection Agency, Research Triangle Park, NC, 1989).

16 Markowitz, G. & Rosner, D. *Lead wars: the politics of science and the fate of America's children*. Vol. 24 (Univ of California Press, 2014).

17 Schwartz, J., Pitcher, H., Levin, R., Ostro, B. & Nichols, A. L. *Costs and benefits of reducing lead in gasoline: Final regulatory impact analysis*. (EPA EPA-230–05–85–006. Washington, DC, 1985).

18 Schwartz, J. Societal benefits of reducing lead exposure. *Environmental Research* **66**, 105-124 (1994).

19 Schwartz, J. *Costs and benefits of reducing lead in gasoline*. Vol. 84 (Office of Policy Analysis, Office of Policy, Planning, and Evaluation, US, 1984).

20 Bellinger, D. C. & Bellinger, A. M. Childhood lead poisoning: the torturous path from science to policy. *The Journal of clinical investigation* **116**, 853-857 (2006).

21 Paulson, J. A. & Brown, M. J. The CDC blood lead reference value for children: Time for a change. *Environmental Health* **18**, 1-3 (2019).

22 Crocetti, A. F., Mushak, P. & Schwartz, J. Determination of numbers of lead-exposed US children by areas of the United States: an integrated summary of a report to the US Congress on childhood lead poisoning. *Environmental Health Perspectives* **89**, 109-120 (1990).

23 Warren, C. *Brush with death: a social history of lead poisoning*. (JHU Press, 2000).

24 Bonnifield, R. S. & Todd, R. Opportunities for the G7 to address the Global Crisis of Lead Poisoning in the 21st Century. (Center for Global Development, Washington, D.C. , 2023).

25 Zou, H., Wang, T., Wang, Z.-L. & Wang, Z. Continuing large-scale global trade and illegal trade of highly hazardous chemicals. *Nature Sustainability*, 1-12 (2023).

26 Ritchie, H. & Roser, M. *Lead Pollution*, <OurWorldInData.org/lead-pollution> (2022).

27 Tatsumoto, M. & Patterson, C. C. Concentrations of common lead in some Atlantic and Mediterranean waters and in snow. *Nature* **199**, 350-352 (1963).

28 Nriagu, J. Sixty years since the report of global lead pollution. (2023).

29 Morrow, P. E., Beiter, H., Amato, F. & Gibb, F. R. Pulmonary retention of lead: An experimental study in man. *Environmental Research* **21**, 373-384 (1980). https://doi.org:https://doi.org/10.1016/0013-9351(80)90040-7

30 Lee, J.-M. *et al.* Coral-based history of lead and lead isotopes of the surface Indian Ocean since the mid-20th century. *Earth and Planetary Science Letters* **398**, 37-47 (2014).

31 Zahran, S., Keyes, C. & Lanphear, B. Leaded aviation gasoline exposure risk and child blood lead levels. *PNAS Nexus* **2** (2023). https://doi.org:10.1093/pnasnexus/pgac285

32 Liang, J. & Mao, J. Source analysis of global anthropogenic lead emissions: their quantities and species. *Environmental Science and Pollution Research* **22**, 7129-7138 (2015).

33 Resongles, E. *et al.* Strong evidence for the continued contribution of lead deposited during the 20th century to the atmospheric environment in London of today. *Proceedings of the National Academy of Sciences* **118**, e2102791118 (2021).

34 Ray, I., Das, R., Chua, S. L. & Wang, X. Seasonal variation of atmospheric Pb sources in Singapore - Elemental and lead isotopic compositions of PM10 as source tracer. *Chemosphere* **307**, 136029 (2022). https://doi.org:https://doi.org/10.1016/j.chemosphere.2022.136029

35 Patterson, C. C. Contaminated and natural lead environments of man. *Archives of Environmental Health: An International Journal* **11**, 344-360 (1965).

36 Young, T. M., Heeraman, D. A., Sirin, G. & Ashbaugh, L. L. Resuspension of Soil as a Source of Airborne Lead near Industrial Facilities and Highways. *Environmental Science & Technology* **36**, 2484-2490 (2002). https://doi.org:10.1021/es015609u

37 Morton-Bermea, O., Rodríguez-Salazar, M. T., Hernández-Alvarez, E., García-Arreola, M. E. & Lozano-Santacruz, R. Lead isotopes as tracers of anthropogenic pollution in urban topsoils of Mexico City. *Geochemistry* **71**, 189-195 (2011).

38 Flegal, A. R., Gallon, C. l., Hibdon, S., Kuspa, Z. E. & Laporte, L. o. F. Declining · but Persistent · Atmospheric Contamination in Central California from the Resuspension of Historic Leaded Gasoline Emissions As Recorded in the Lace Lichen (Ramalina menziesii Taylor) from 1892 to 2006. *Environmental science & technology* **44**, 5613-5618 (2010).

39 Harris, A. R. & Davidson, C. I. The role of resuspended soil in lead flows in the California South Coast Air Basin. *Environmental science & technology* **39**, 7410-7415 (2005).

40 Sturges, W. & Barrie, L. Lead 206/207 isotope ratios in the atmosphere of North America as tracers of US and Canadian emissions. *Nature* **329**, 144-146 (1987).

41 Erel, Y. *et al.* Transboundary atmospheric lead pollution. *Environmental science & technology* **36**, 3230-3233 (2002).

42 Ewing, S. A. *et al.* Pb isotopes as an indicator of the Asian contribution to particulate air pollution in urban California. *Environmental science & technology* **44**, 8911-8916 (2010).

43 Koffman, B. G. *et al.* Provenance of Anthropogenic Pb and Atmospheric Dust to Northwestern North America. *Environmental Science & Technology* **56**, 13107-13118 (2022). https://doi.org:10.1021/acs.est.2c03767

44 Zurbrick, C. M., Gallon, C. & Flegal, A. R. Historic and industrial lead within the Northwest Pacific Ocean evidenced by lead isotopes in seawater. *Environmental Science & Technology* **51**, 1203-1212 (2017).

45 Chen, M. *et al.* Dissolved lead (Pb) concentrations and Pb isotope ratios along the East China Sea and Kuroshio transect ‐ evidence for isopycnal transport and particle exchange. *Journal of Geophysical Research: Oceans*, e2022JC019423 (2023).

46 Chen, Q. & Taylor, D. Transboundary atmospheric pollution in Southeast Asia: current methods, limitations and future developments. *Critical Reviews in Environmental Science and Technology* **48**, 997-1029 (2018).

47 Zhang, Q. *et al.* Transboundary health impacts of transported global air pollution and international trade. *Nature* **543**, 705-709 (2017). https://doi.org:10.1038/nature21712

48 Weiss, D. *et al.* Spatial and temporal evolution of lead isotope ratios in the North Atlantic Ocean between 1981 and 1989. *Journal of Geophysical Research: Oceans* **108** (2003).

49 Bridgestock, L. *et al.* Return of naturally sourced Pb to Atlantic surface waters. *Nature Communications* **7**, 12921 (2016).

50 Nozaki, Y., Thomson, J. & Turekian, K. The distribution of 210Pb and 210Po in the surface waters of the Pacific Ocean. *Earth and Planetary Science Letters* **32**, 304-312 (1976).

51 Lee, J.-M. *et al.* Impact of anthropogenic Pb and ocean circulation on the recent distribution of Pb isotopes in the Indian Ocean. *Geochimica et Cosmochimica Acta* **170**, 126-144 (2015).

52 Echegoyen, Y. *et al.* Recent distribution of lead in the Indian Ocean reflects the impact of regional emissions. *Proceedings of the National Academy of Sciences* **111**, 15328-15331 (2014).

53 Pinedo-González, P., West, A. J., Tovar-Sanchez, A., Duarte, C. M. & Sañudo-Wilhelmy, S. A. Concentration and isotopic composition of dissolved Pb in surface waters of the modern global ocean. *Geochimica et Cosmochimica Acta* **235**, 41-54 (2018).

54 Noble, A. E. *et al.* Dynamic variability of dissolved Pb and Pb isotope composition from the US North Atlantic GEOTRACES transect. *Deep Sea Research Part II: Topical Studies in Oceanography* **116**, 208-225 (2015).

55 Olivelli, A. *et al.* Decline of anthropogenic lead in South Atlantic Ocean surface waters from 1990 to 2011: New constraints from concentration and isotope data. *Marine Pollution Bulletin* **189**, 114798 (2023).

56 Boye, M. *et al.* Distributions of dissolved trace metals (Cd, Cu, Mn, Pb, Ag) in the southeastern Atlantic and the Southern Ocean. *Biogeosciences* **9**, 3231-3246 (2012).

57 GEOTRACES Intermediate Data Product Group. The GEOTRACES Intermediate Data Product 2021 (IDP2021). *NERC EDS British Oceanographic Data Centre NOC* (2021).

58 Henderson, G. M. & Maier-Reimer, E. Advection and removal of 210Pb and stable Pb isotopes in the oceans: a general circulation model study. *Geochimica et Cosmochimica Acta* **66**, 257-272 (2002).

59 Jiang, S., Zhang, J., Zhang, R., Xue, Y. & Zheng, W. Dissolved lead in the East China Sea with implications for impacts of marginal seas on the open ocean through cross ‐ shelf exchange. *Journal of Geophysical Research: Oceans* **123**, 6004-6018 (2018).

60 Mitra, S., Sarkar, S. K., Raja, P., Biswas, J. K. & Murugan, K. Dissolved trace elements in Hooghly (Ganges) River Estuary, India: Risk assessment and implications for management. *Marine pollution bulletin* **133**, 402-414 (2018).

61 Chen, M. *et al.* Boundary exchange completes the marine Pb cycle jigsaw. *Proceedings of the National Academy of Sciences* **120**, e2213163120 (2023).

62 Rusiecka, D. *et al.* Anthropogenic signatures of lead in the Northeast Atlantic. *Geophysical Research Letters* **45**, 2734-2743 (2018).

63 Mielke, H. W., Gonzales, C. R., Powell, E. T. & Egendorf, S. P. Lead in Air, Soil, and Blood: Pb Poisoning in a Changing World. *International Journal of Environmental Research and Public Health* **19**, 9500 (2022).

64 Dia, A., Chauvel, C., Bulourde, M. & Gérard, M. Eolian contribution to soils on Mount Cameroon: Isotopic and trace element records. *Chemical Geology* **226**, 232-252 (2006).

65 Wang, Z. *et al.* Legacy of anthropogenic lead in urban soils: Co-occurrence with metal (loids) and fallout radionuclides, isotopic fingerprinting, and in vitro bioaccessibility. *Science of The Total Environment* **806**, 151276 (2022).

66 Kylander, M. E. *et al.* Refining the pre-industrial atmospheric Pb isotope evolution curve in Europe using an 8000 year old peat core from NW Spain. *Earth and Planetary Science Letters* **240**, 467-485 (2005).

67 Erel, Y., Veron, A. & Halicz, L. Tracing the transport of anthropogenic lead in the atmosphere and in soils using isotopic ratios. *Geochimica et Cosmochimica Acta* **61**, 4495-4505 (1997).

68 Teutsch, N., Erel, Y., Halicz, L. & Banin, A. Distribution of natural and anthropogenic lead in Mediterranean soils. *Geochimica et Cosmochimica Acta* **65**, 2853-2864 (2001).

69 Emmanuel, S. & Erel, Y. Implications from concentrations and isotopic data for Pb partitioning processes in soils. *Geochimica et Cosmochimica Acta* **66**, 2517-2527 (2002).

70 Harrison, R. M., Laxen, D. P. & Wilson, S. J. Chemical associations of lead, cadmium, copper, and zinc in street dusts and roadside soils. *Environmental Science & Technology* **15**, 1378-1383 (1981).

71 Margenat, A. *et al.* Occurrence and human health implications of chemical contaminants in vegetables grown in peri-urban agriculture. *Environment international* **124**, 49-57 (2019).

72 EIA US. *Petroleum & other liquids*, <https://www.eia.gov/petroleum/data.php> (2021).

73 Chen, M. *et al.* Monsoonal variations of lead (Pb) in coastal waters around Singapore. *Marine Pollution Bulletin* **179**, 113654 (2022).

74 Hassel, D. *et al.* Exhaust emission factors for motor vehicles in the Federal Republic of Germany for the reference year 1990. *Final Report of a Study Carried out on Behalf of the Federal Environmental Protection Agency, UFOPLAN*, 05 (1993).

75 Li, Q., Cheng, H., Zhou, T., Lin, C. & Guo, S. The estimated atmospheric lead emissions in China, 1990–2009. *Atmospheric Environment* **60**, 1-8 (2012).

76 The international energy agency. *Data and Statistics*, <https://www.iea.org/> (n.d.).

77 Singh, A. K. & Singh, M. Lead decline in the Indian environment resulting from the petrol-lead phase-out programme. *Science of the Total Environment* **368**, 686-694 (2006).

78 Mukai, H. *et al.* Characterization of sources of lead in the urban air of Asia using ratios of stable lead isotopes. *Environmental Science & Technology* **27**, 1347-1356 (1993).

79 Hirota, K. Review of lead phase out for air quality improvement in the third world cities lessons from Thailand and Indonesia. *Studies in Regional Science* **36**, 527-541 (2006).

80 Hosono, T. *et al.* Decline in heavy metal contamination in marine sediments in Jakarta Bay, Indonesia due to increasing environmental regulations. *Estuarine, Coastal and Shelf Science* **92**, 297-306 (2011).

81 Kondo, A. *et al.* Impacts of converting from leaded to unleaded gasoline on ambient lead concentrations in Jakarta metropolitan area. *Journal of Environmental Sciences* **19**, 709-713 (2007).

82 Supat, W. in *Better Air Quality in Asian and Pacific Rim Cities 2002 Workshop (BAQ 2002).*

83 Balce, G. R. Overview of Regional Fuel Market and Fuel Quality Standards in ASEAN presentation slides *ASEAN Centre for Energy 2001* (2001).

84 WorldBank. *An overnight sucess: Vietnam's switch to unleaded gasoline*, <https://openknowledge.worldbank.org/handle/10986/19894> (2002).

85 Fitchett, D., Jaspersen, F., Pfeffermann, G., Karmokolias, I. & Glen, J. An overnight sucess: Vietnam's switch to unleaded gasoline. (2002).

86 Environmental Management Bureau (Philippine). *National Air Quality Status Report*. (2002).

87 Riddell, T. J. *et al.* Elevated blood-lead levels among children living in the rural Philippines. *Bulletin of the World Health Organization* **85**, 674-680 (2007).

88 Carmela C. Manocan. Philippine National Standards and its Importance. *Energy Consumers and Stakeholders' Conference 2018* (2018).

89 Onursal, B. & Gautam, S. *Vehicular air pollution: experiences from seven Latin American urban centers*. Vol. 373 (World Bank Publications, 1997).

90 European Environmental Agency. Air Pollutant Emissions Data Viewer (Gothenburg Protocol, LRTAP Convention) 1990–2020.

91 Murray, C. J. *et al.* Global burden of 87 risk factors in 204 countries and territories, 1990–2019: a systematic analysis for the Global Burden of Disease Study 2019. *The lancet* **396**, 1223-1249 (2020).

92 Crump, K. S., Van Landingham, C., Bowers, T. S., Cahoy, D. & Chandalia, J. K. A statistical reevaluation of the data used in the Lanphear et al. pooled-analysis that related low levels of blood lead to intellectual deficits in children. *Critical reviews in toxicology* **43**, 785-799 (2013).

93 GBD Risk Factors Collaborators. The unfulfilled promise of prevention: the global burden of 87 risk factors, 1990–2019; a systematic analysis for the Global Burden of Disease Study 2019. *The Lancet* **396**, 1223-1249 (2020).

94 WorldBank. *World Development Indicators*, <https://data.worldbank.org/> (2024).

95 Klemick, H., Mason, H. & Sullivan, K. Superfund cleanups and children's lead exposure. *Journal of environmental economics and management* **100**, 102289 (2020).

96 Montenegro, C. E. & Patrinos, H. A. A data set of comparable estimates of the private rate of return to schooling in the world, 1970–2014. *International Journal of Manpower* (2021).

97 Mincer, J. Investment in human capital and personal income distribution. *Journal of political economy* **66**, 281-302 (1958).

98 Annest, J. L. *et al.* Chronological Trend in Blood Lead Levels between 1976 and 1980. *New England Journal of Medicine* **308**, 1373-1377 (1983). https://doi.org:10.1056/nejm198306093082301

99 Brown, M. J. & Margolis, S. Lead in drinking water and human blood lead levels in the United States. (2012).

100 The Nation. *8,500 Years of Lead - 79 Years of leaded gasoline*, <link.gale.com/apps/doc/A60025338/ITOF?u=umuser&sid=bookmark-ITOF&xid=1205cf9e.> (2000).

101 Oudijk, G. The rise and fall of organometallic additives in automotive gasoline. *Environmental Forensics* **11**, 17-49 (2010).

102 Lee, K. *et al.* Isotopic signatures for natural versus anthropogenic Pb in high-altitude Mt. Everest ice cores during the past 800 years. *Science of the Total Environment* **412**, 194-202 (2011).

103 Shotyk, W., Blaser, P., Grünig, A. & Cheburkin, A. A new approach for quantifying cumulative, anthropogenic, atmospheric lead deposition using peat cores from bogs: Pb in eight Swiss peat bog profiles. *Science of the Total Environment* **249**, 281-295 (2000).

104 Wang, C., Wang, J., Zhao, Y. & Zhong, C. The vertical migration and speciation of the Pb in the paddy soil: a case study of the Yangtze River Delta, China. *Environmental research* **179**, 108741 (2019).

105 McConnell, J. R. *et al.* Hemispheric-scale heavy metal pollution from South American and Australian mining and metallurgy during the Common Era. *Science of The Total Environment* **912**, 169431 (2024). https://doi.org:https://doi.org/10.1016/j.scitotenv.2023.169431

106 Legrand, M. *et al.* Cadmium Pollution From Zinc-Smelters up to Fourfold Higher Than Expected in Western Europe in the 1980s as Revealed by Alpine Ice. *Geophysical Research Letters* **47**, e2020GL087537 (2020). https://doi.org:https://doi.org/10.1029/2020GL087537

107 Hashimoto, Y., Sekine, Y., Kim, H. K., Chen, Z. L. & Yang, Z. M. Atmospheric fingerprints of east Asia, 1986–1991. An urgent record of aerosol analysis by the jack network. *Atmospheric Environment* **28**, 1437-1445 (1994).

108 Bollhöfer, A. & Rosman, K. Isotopic source signatures for atmospheric lead: the Northern Hemisphere. *Geochimica et Cosmochimica Acta* **65**, 1727-1740 (2001).

109 He, K. *et al.* The characteristics of PM2. 5 in Beijing, China. *Atmospheric Environment* **35**, 4959-4970 (2001).

110 Okuda, T. *et al.* Trends in hazardous trace metal concentrations in aerosols collected in Beijing, China from 2001 to 2006. *Chemosphere* **72**, 917-924 (2008).

111 Sun, Y., Zhuang, G., Zhang, W., Wang, Y. & Zhuang, Y. Characteristics and sources of lead pollution after phasing out leaded gasoline in Beijing. *Atmospheric Environment* **40**, 2973-2985 (2006).

112 Cai, J. *et al.* Source apportionment of Pb-containing particles in Beijing during January 2013. *Environmental Pollution* **226**, 30-40 (2017).

113 Wu, X., Chen, B., Wen, T., Habib, A. & Shi, G. Concentrations and chemical compositions of PM10 during hazy and non-hazy days in Beijing. *Journal of Environmental Sciences* **87**, 1-9 (2020).

114 Tao, Z. *et al.* Atmospheric lead pollution in a typical megacity: Evidence from lead isotopes. *Science of The Total Environment* **778**, 145810 (2021).

115 Tao, Z. *et al.* Slight transition in Chinese atmospheric Pb isotopic fingerprinting due to increasing foreign Pb. *Environmental Pollution* **323**, 121296 (2023).

116 Balachandran, S., Meena, B. R. & Khillare, P. Particle size distribution and its elemental composition in the ambient air of Delhi. *Environment international* **26**, 49-54 (2000).

117 Bandhu, H. *et al.* Elemental composition and sources of air pollution in the city of Chandigarh, India, using EDXRF and PIXE techniques. *Nuclear Instruments and Methods in Physics Research Section B: Beam Interactions with Materials and Atoms* **160**, 126-138 (2000).

118 Bhuyan, P., Deka, P., Prakash, A., Balachandran, S. & Hoque, R. R. Chemical characterization and source apportionment of aerosol over mid Brahmaputra Valley, India. *Environmental Pollution* **234**, 997-1010 (2018).

119 Bikkina, S., Sarin, M. & Chinni, V. Atmospheric 210Pb and anthropogenic trace metals in the continental outflow to the Bay of Bengal. *Atmospheric Environment* **122**, 737-747 (2015).

120 Boreddy, S. K., Hegde, P. & Aswini, A. Geochemical characteristics of trace elements in size-resolved coastal urban aerosols associated with distinct air masses over tropical peninsular India: Size distributions and source apportionment. *Science of The Total Environment* **763**, 142967 (2021).

121 Mouli, P. C., Mohan, S. V., Balaram, V., Kumar, M. P. & Reddy, S. J. A study on trace elemental composition of atmospheric aerosols at a semi-arid urban site using ICP-MS technique. *Atmospheric Environment* **40**, 136-146 (2006).

122 Chelani, A., Gajghate, D. & Hasan, M. Airborne toxic metals in air of Mumbai city, India. *Bulletin of environmental contamination and toxicology* **66**, 196-205 (2001).

123 Chinnam, N., Dey, S., Tripathi, S. & Sharma, M. Dust events in Kanpur, northern India: Chemical evidence for source and implications to radiative forcing. *Geophysical Research Letters* **33** (2006).

124 Das, M., Maiti, S. K. & Mukhopadhyay, U. Distribution of PM 2.5 and PM 10-2.5 in PM 10 fraction in ambient air due to vehicular pollution in Kolkata megacity. *Environmental Monitoring and Assessment* **122**, 111-123 (2006).

125 Das, R., Mohtar, A. T. B. M., Rakshit, D., Shome, D. & Wang, X. Sources of atmospheric lead (Pb) in and around an Indian megacity. *Atmospheric Environment* **193**, 57-65 (2018).

126 Dubey, B., Pal, A. K. & Singh, G. Trace metal composition of airborne particulate matter in the coal mining and non–mining areas of Dhanbad Region, Jharkhand, India. *Atmospheric Pollution Research* **3**, 238-246 (2012).

127 Gaghate, D. & Hasan, M. Ambient lead levels in urban areas. *Bulletin of environmental contamination and toxicology* **62**, 403-408 (1999).

128 Gajghate, D. & Bhanarkar, A. Characterisation of particulate matter for toxic metals in ambient air of Kochi city, India. *Environmental Monitoring and Assessment* **102**, 119-129 (2005).
129 Gajghate, D., Talwar, B., Pipalatkar, P. & Pustode, T. Chemical characterization of PM 10 for metals in ambient air of Chennai, India. *Journal of hazardous, toxic, and radioactive waste* **16**, 169-174 (2012).
130 Gaonkar, C. V., Kumar, A., Matta, V. M. & Kurian, S. Assessment of crustal element and trace metal concentrations in atmospheric particulate matter over a coastal city in the Eastern Arabian Sea. *Journal of the Air & Waste Management Association* **70**, 78-92 (2020).
131 Gokhale, S. & Patil, R. Size distribution of aerosols (PM 10) and lead (Pb) near traffic intersections in Mumbai (India). *Environmental monitoring and assessment* **95**, 311-324 (2004).
132 Gummeneni, S., Yusup, Y. B., Chavali, M. & Samadi, S. Source apportionment of particulate matter in the ambient air of Hyderabad city, India. *Atmospheric Research* **101**, 752-764 (2011).
133 Jain, S., Sharma, S., Vijayan, N. & Mandal, T. Seasonal characteristics of aerosols (PM2. 5 and PM10) and their source apportionment using PMF: a four year study over Delhi, India. *Environmental Pollution* **262**, 114337 (2020).
134 Jena, S., Perwez, A. & Singh, G. Trace element characterization of fine particulate matter and assessment of associated health risk in mining area, transportation routes and institutional area of Dhanbad, India. *Environmental geochemistry and health* **41**, 2731-2747 (2019).
135 Joseph, A. E., Unnikrishnan, S. & Kumar, R. Chemical characterization and mass closure of fine aerosol for different land use patterns in Mumbai city. *Aerosol and Air Quality Research* **12**, 61-72 (2012).
136 Kar, S., Maity, J. P., Samal, A. C. & Santra, S. C. Metallic components of traffic-induced urban aerosol, their spatial variation, and source apportionment. *Environmental monitoring and assessment* **168**, 561-574 (2010).
137 Karar, K. & Gupta, A. Source apportionment of PM10 at residential and industrial sites of an urban region of Kolkata, India. *Atmospheric Research* **84**, 30-41 (2007).
138 Khare, P. & Baruah, B. P. Elemental characterization and source identification of PM2. 5 using multivariate analysis at the suburban site of North-East India. *Atmospheric Research* **98**, 148-162 (2010).
139 Khillare, P., Balachandran, S. & Meena, B. R. Spatial and temporal variation of heavy metals in atmospheric aerosol of Delhi. *Environmental Monitoring and Assessment* **90**, 1-21 (2004).
140 Khillare, P. S. & Sarkar, S. Airborne inhalable metals in residential areas of Delhi, India: distribution, source apportionment and health risks. *Atmospheric pollution research* **3**, 46-54 (2012).
141 Kulshrestha, A., Satsangi, P. G., Masih, J. & Taneja, A. Metal concentration of PM2. 5 and PM10 particles and seasonal variations in urban and rural environment of Agra, India. *Science of the Total Environment* **407**, 6196-6204 (2009).
142 Kumar, A. V., Patil, R. & Nambi, K. Source apportionment of suspended particulate matter at two traffic junctions in Mumbai, India. *Atmospheric Environment* **35**, 4245-4251 (2001).
143 Kumar, S. *et al.* Tracing dust transport from Middle-East over Delhi in March 2012 using metal and lead isotope composition. *Atmospheric Environment* **132**, 179-187 (2016).
144 Mehra, M., Zirzow, F., Ram, K. & Norra, S. Geochemistry of PM2. 5 aerosols at an urban site, Varanasi, in the Eastern Indo-Gangetic Plain during pre-monsoon season. *Atmospheric Research* **234**, 104734 (2020).
145 Mitra, A. *et al.* Lead Isotope evidence for enhanced anthropogenic particle transport to the Himalayas during summer months. *Environmental Science & Technology* **55**, 13697-13708 (2021).
146 Mishra, V. K. & Padmanabhamutry, B. Performance evaluation of CALINE3, CAL3QHC and PART5 in predicting lead concentration in the atmosphere over Delhi. *Atmospheric Environment* **37**, 3077-3089 (2003).

147 Negi, B., Sadasivan, S. & Mishra, U. Aerosol composition and sources in urban areas in India. *Atmospheric Environment (1967)* **21**, 1259-1266 (1967).

148 Panda, S., Mallik, C., Nath, J., Das, T. & Ramasamy, B. A study on variation of atmospheric pollutants over Bhubaneswar during imposition of nationwide lockdown in India for the COVID-19 pandemic. *Air Quality, Atmosphere & Health* **14**, 97-108 (2021).

149 Pandey, P. *et al.* Temporal distribution of fine particulates (PM2. 5, PM10), potentially toxic metals, PAHs and Metal-bound carcinogenic risk in the population of Lucknow City, India. *Journal of Environmental Science and Health, Part A* **48**, 730-745 (2013).

150 Pant, P. *et al.* Characterization of ambient PM2. 5 at a pollution hotspot in New Delhi, India and inference of sources. *Atmospheric environment* **109**, 178-189 (2015).

151 Perrino, C. *et al.* Chemical characterization of atmospheric PM in Delhi, India, during different periods of the year including Diwali festival. *Atmospheric Pollution Research* **2**, 418-427 (2011).

152 Pervez, S. *et al.* Chemical speciation of aerosols and air quality degradation during the festival of lights (Diwali). *Atmospheric Pollution Research* **7**, 92-99 (2016).

153 Police, S., Sahu, S. K. & Pandit, G. G. Chemical characterization of atmospheric particulate matter and their source apportionment at an emerging industrial coastal city, Visakhapatnam, India. *Atmospheric Pollution Research* **7**, 725-733 (2016).

154 Sah, D., Verma, P. K., Kumari, K. M. & Lakhani, A. Chemical fractionation of heavy metals in fine particulate matter and their health risk assessment through inhalation exposure pathway. *Environmental geochemistry and health* **41**, 1445-1458 (2019).

155 Sen, I. S., Bizimis, M., Tripathi, S. N. & Paul, D. Lead isotopic fingerprinting of aerosols to characterize the sources of atmospheric lead in an industrial city of India. *Atmospheric Environment* **129**, 27-33 (2016).

156 Shridhar, V., Khillare, P., Agarwal, T. & Ray, S. Metallic species in ambient particulate matter at rural and urban location of Delhi. *Journal of Hazardous Materials* **175**, 600-607 (2010).

157 M, A. S., V.G, G., N.G, V. & Mohan, M. Trace gases and PM2.5-bound metal abundance over a tropical urban environment, South India. *Journal of Atmospheric Chemistry* **78**, 193-208 (2021). https://doi.org:10.1007/s10874-021-09420-1

158 Singh, R. & Sharma, B. S. Composition, seasonal variation, and sources of PM 10 from world heritage site Taj Mahal, Agra. *Environmental Monitoring and Assessment* **184**, 5945-5956 (2012).

159 Soni, A., Kumar, U., Prabhu, V. & Shridhar, V. Characterization, source apportionment and carcinogenic risk assessment of atmospheric particulate matter at Dehradun, situated in the Foothills of Himalayas. *Journal of Atmospheric and Solar-Terrestrial Physics* **199**, 105205 (2020).

160 Sudheer, A. & Rengarajan, R. Atmospheric mineral dust and trace metals over urban environment in western India during winter. *Aerosol and Air Quality Research* **12**, 923-933 (2012).

161 Yadav, S. & Satsangi, P. G. Characterization of particulate matter and its related metal toxicity in an urban location in South West India. *Environmental monitoring and assessment* **185**, 7365-7379 (2013).

162 Prakash, J. *et al.* Chemical characterization and quantitativ e assessment of source-specific health risk of trace metals in PM 1.0 at a road site of Delhi, India. *Environmental Science and Pollution Research* **25**, 8747-8764 (2018).

163 Petit, D., Veron, A., Flament, P., Deboudt, K. & Poirier, A. Review of pollutant lead decline in urban air and human blood: A case study from northwestern Europe. *Comptes Rendus Geoscience* **347**, 247-256 (2015).

164 US Environmental Protection Agency. *Lead Trends*, <https://www.epa.gov/air-trends/lead-trends> (2023).

165 Singapore National Environmental Agency. Environmental Protection Division Report 2011. (2011).

166 Singapore National Environmental Agency. State of the Environment: Air & Water Quality. A report by NEA's Environmental Monitoring and Modelling Division. (2021).

167 Das, R. *et al.* Suspension of Crustal Materials from Wildfire in Indonesia as Revealed by Pb Isotope Analysis. *ACS Earth and Space Chemistry* **7**, 379-387 (2023).

168 Bi, X.-Y. *et al.* Lead isotopic compositions of selected coals, Pb/Zn ores and fuels in China and the application for source tracing. *Environmental science & technology* **51**, 13502-13508 (2017).

169 Ministry of Ecology and Environment of China. *国务院办公厅关于限期停止生产销售使用车用含铅汽油的通知 (in Chinese)*, <https://www.mee.gov.cn/zcwj/gwywj/201811/t20181129_676365.shtml> (1998).
